# Interleukin‐39 is a Prognostic Biomarker and Therapy Target for Sepsis

**DOI:** 10.1002/advs.77663

**Published:** 2026-09-10

**Authors:** Feng‐zhi Zhang, Hai‐jun Liu, Yan Yue, Jun‐jie Gao, Jin Zhang, Wen‐wu Zhang, Yun‐long Zhang, Wei‐hua Lu, Sheng‐wei Jin, Pu‐hong Zhang

**Affiliations:** ^1^ Department of Critical Care Medicine Anhui Province Clinical Research Center for Critical Respiratory Medicine The First Affiliated Hospital of Wannan Medical University (Yijishan Hospital) Wuhu Anhui Province People's Republic of China; ^2^ Department of Clinical Laboratory The Second Affiliated Hospital of Wannan Medical University Wuhu Anhui Province People's Republic of China; ^3^ Department of Anesthesia and Critical Care The Second Affiliated Hospital and Yuying Children's Hospital Key Laboratory of Pediatric Anesthesiology Ministry of Education Precision Anesthesiology Key Laboratory of Zhejiang Province Wenzhou Medical University Wenzhou People's Republic of China

**Keywords:** GP130, IL‐39, inflammatory factors, macrophage, sepsis

## Abstract

Sepsis remains a global health crisis with high mortality, due to a paucity of reliable diagnostic markers for accurate risk stratification and precision management. Interleukin‐39 (IL‐39), a novel immunomodulatory cytokine, plays an important role in regulating the pathophysiology of immunity, metabolism, and et al. Here, we observed significantly elevated serum IL‐39 levels in septic patients at admission compared with non‐sepsis ICU patients and healthy controls across two independent cohorts. Furthermore, circulating IL‐39 concentrations could predict 28‐days survival in patients with sepsis. Single‐cell sequencing demonstrated that elevated IL‐39 was derived from macrophages during sepsis. Meanwhile, supplementation of IL‐39, via either recombinant protein (rmIL‐39) administration or macrophage‐specific AAV‐mediated overexpression, profoundly aggravates multiple organ dysfunction and damage in septic mice. Consistently, macrophage IL‐39 deficiency, via either macrophage‐conditional knockout or macrophage‐specific AAV‐mediated silence, protects against sepsis. Mechanistically, IL‐39 engages GP130 to trigger MAPK/P38 signaling, driving pro‐inflammatory cytokine production. Consequently, macrophage‐specific GP130 deletion abrogates IL‐39‐induced sepsis aggravation. Finally, pharmaceutical inhibition of IL‐39 with neutralizing antibodies can protect against sepsis. Together, these results highlight the dual clinical utility of IL‐39, as both a robust biomarker with independent prognostic value for sepsis patient stratification and a promising therapeutic strategy for sepsis.

## Graphical abstract

Sepsis triggers a significant increase in IL‐39 production, with circulating levels markedly elevated and positively correlated with disease severity and poor clinical prognosis in sepsis patients. Mechanistically, macrophage‐derived IL‐39 activates the GP130 signaling pathway via a ligand‐receptor interaction, thereby fueling the pro‐inflammatory expression that exacerbates hyper‐inflammation, organ dysfunction, and tissue damage in sepsis.

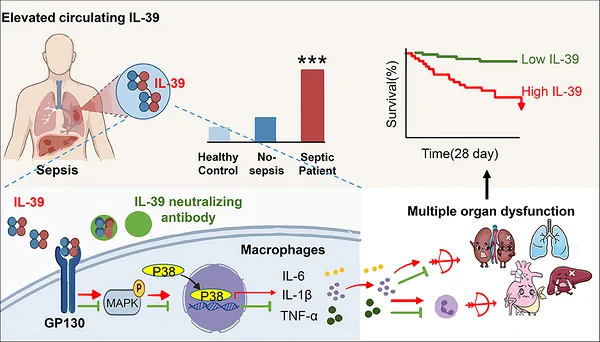

## 1 Introduction

Sepsis is defined as a life‐threatening condition involving systemic organ failure, precipitated by a dysfunctional immune response to pathogens. The central pathophysiology of this syndrome involves a profoundly complex imbalance in host inflammatory signaling, which manifests as a pathological coexistence of hyper‐inflammation and subsequent immune paralysis [1]. Infection control and organ functional support are the main interventions for sepsis, but there remain no specialized pharmacological agents validated for the therapy of sepsis [2]. Host‐derived immune regulatory molecules possess a critical impact on modulating innate immune signaling and function as vital immune targets for the clinical assessment of sepsis and host‐directed therapy [3, 4, 5]. However, despite extensive research into immune regulatory factors aimed at finding biologics that can modify the immune profile in sepsis, no notable favorable outcomes or survival‐improving benefits have been demonstrated in clinical practice [6, 7]. Thus, the development of novel therapeutic biomarkers is essential to facilitate patient identification and function as targets for host‐directed interventions.

Interleukin‐39 (IL‐39) cytokines, a recently discovered member of the IL‐12 family, is composed of the EBI3 (Epstein‐Barr virus‐induced gene 3) subunit and the IL‐23p19 subunit and is widely expressed in various tissues such as the lungs, skin, and kidneys [8, 9, 10, 11]. Recent studies have revealed IL‐39 is produced and secreted by macrophages, B cells, dendritic cells, and some epithelial cells under inflammatory stimulation and plays a critical regulatory role in the pathological processes of myasthenia gravis [12], diabetes mellitus [13, 14], lung cancer [15], tuberculous pleurisy [16], inflammatory bowel disease [17], rheumatoid arthritis [18], periodontitis [19], and other chronic inflammation [20]. Nevertheless, the role and mechanism of IL‐39 signaling in the pathophysiology of sepsis remain undetermined. Here, we determined that IL‐39 is a potential candidate prognostic biomarker for sepsis. Furthermore, we found that elevated IL‐39, derived from macrophages, exacerbates hyper‐inflammation and multiple organ damage in polymicrobial sepsis, and highlighted that IL‐39 inhibition is a potential treatment strategy for sepsis.

## 2 Methods and Materials

### 2.1 Study Design

This study aimed to elucidate the function and clinical significance of IL‐39 in sepsis.

The prognostic potential of circulating IL‐39 levels was evaluated in two distinct, independently enrolled human sepsis cohorts. The discovery cohort was from the First Affiliated Hospital of Wannan Medical University (Anhui cohort) and the validation cohort from the Second Affiliated Hospital and Yuying Children's Hospital of Wenzhou Medical University (Zhejiang cohort). All patients fulfilled the diagnostic definitions stipulated by the Sepsis‐3 guidelines [1]. The Anhui cohort comprised 90 sepsis patients. The Zhejiang cohort comprised 122 sepsis patients. The cohort study employed strict exclusion criteria: minors, pregnant, breastfeeding, autoimmune diseases, HIV infection, and organ transplantation as well as patients currently receiving immunosuppressive therapy [21].

We employed in vivo in mice models (polymicrobial sepsis) and in vitro cell culture models to evaluate the therapeutic potential of IL‐39 in sepsis and elucidate the molecular mechanisms by which it governs inflammation and multiple organ damage. For all animal studies, mice were randomized into treatment or genotype‐dependent groups with a target statistical power of 80% (α = 0.05), utilizing sample sizes consistent with established field standards. The primary experimental endpoint was survival. In vitro assay parameters and sample sizes were determined based on preliminary data and peer‐reviewed literature. To ensure unbiased results, all histological, and cellular analyses were conducted under blinded conditions prior to quantification. No data were excluded from the analysis; specific sample sizes and experimental replicates are detailed in the respective figure legends and Supporting Information and Methods.

### 2.2 Statistical Analysis

Comprehensive individual‐level data are provided in Supporting Information. Qualitative variables were analyzed by the chi‐square test or Fisher's exact test, while comparisons of quantitative data distributions were performed using the Mann‐Whitney U test or Kruskal‐Wallis test; where applicable, Dunn's multiple comparison post‐hoc tests were used to determine statistical significance. Correlation was quantified using the Spearman correlation coefficient (*r*). To assess the predictive and prognostic performance of IL‐39 alongside conventional markers, receiver operating characteristic (ROC) analysis was performed, with area under the curve (AUC) values ​​calculated for IL‐39, PCT, CRP, and SOFA scores.

To stratify survival outcomes, patients were categorized into high vs. low serum IL‐39 groups based on a threshold derived from ROC curve analysis. This optimal cutoff was identified using the minimum *d* calculation, which determines the point on the ROC curve with the shortest distance to the coordinates, thereby maximizing both sensitivity and specificity. Utilizing a threshold value of 32.38 pg/mL as the optimal discriminatory point, we classified patients as having high (≥32.38 pg/mL) vs. low (<32.38 pg/mL) serum IL‐39 concentrations. The prognostic value of serum IL‐39 levels for 28‐day mortality was rigorously assessed using univariate and multivariate Cox regression models. In addition, we plotted Kaplan‐Meier survival curves and their 95% confidence intervals for both cohorts to illustrate survival differences, and performed analysis using the log‐rank test. All statistical analyses were performed using SPSS (version 26.0; Armonk, New York, USA) and GraphPad Prism software (version 10.0; La Jolla, CA, USA). Statistical significance was defined as a two‐sided p‐value < 0.05. All experiments were independently repeated at least three times to ensure the reproducibility and statistical validity of the results. Details of the statistical tests used for each dataset are documented in the corresponding legends and Supporting Information.

## 3 Results

### 3.1 Circulating IL‐39 Levels are Markedly Elevated and Positively Associated with Severity in Patients with Sepsis

**FIGURE 1:**
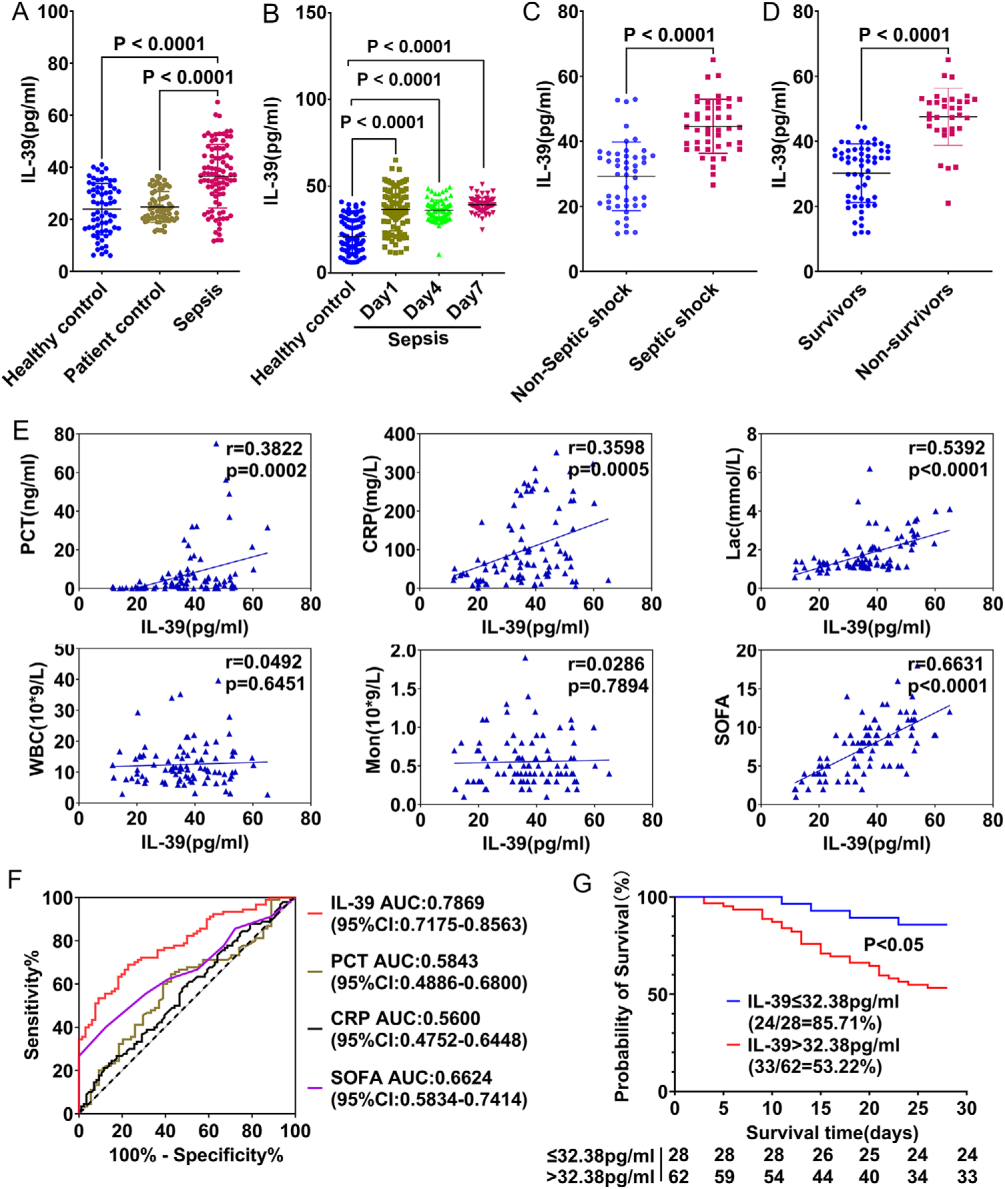
Elevated IL‐39 levels were associated with worse survival for the patients with sepsis in the Anhui discovery cohort. (A) Levels of L‐39 were detected in serum samples from healthy controls (*n* = 57), patient controls (*n* = 66), and sepsis patients (*n* = 90) using a “double‐antibody sandwich” enzyme‐linked immunosorbent assay (ELISA). (B) Dynamic detection of plasma IL‐39 levels of sepsis patients at days 1, 4, and 7 compared with healthy controls. (C) Plasma levels of IL‐39 in septic shock patients (*n* = 43) and non‐shock patients (*n* = 47). (D) Plasma levels of IL‐39 in septic survivors (*n* = 57) and non‐survivors (*n* = 33). (E) Correlations between IL‐39 levels and PCT concentrations, CRP concentrations, Lau concentrations, WBC counts, and Mon counts as well as SOFA scores in septic patients. (F) ROC curves for IL‐39, PCT, CRP, and SOFA at admission in predicting 28‐day mortality of patients with sepsis. (G) Kaplan‐ Meier survival curves of 90 patients with sepsis based on the IL‐39 cutoff value (32.38 pg/mL) on the day of ICU admission. Data are represented as mean ± SD (A–D) and determined by the Kruskal‐Wallis test followed by Dun multiple comparisons posttest in (A, B), the two‐tailed unpaired Student's t test in (C, D). Spearman correlation coefficient (r) in (E); Kaplan‐ Meier analysis followed by log‐rank test in (F). log‐ rank test in (G); ns, not significant.

To explore the clinical characteristics of IL‐39 in patients with sepsis, serum levels of IL‐39 were measured by “double‐antibody sandwich” enzyme‐linked immunosorbent assay (ELISA). The discovery cohort (Anhui) comprised 90 patients with sepsis, 65 patients with no sepsis (patient controls), and 30 healthy volunteers (Table S1). The 28‐day mortality was 36.66% in the Anhui cohort patients (57 survivors, 33 non‐survivors). In the Anhui cohort, sepsis patients had higher serum levels of IL‐39 at admission compared with non‐sepsis patients or healthy volunteers (*p* < 0.0001, Figure 1A). Serum IL‐39 in sepsis patients persisted at high levels until day 7 (*p* < 0.0001, Figure 1B). In addition, IL‐39 levels were higher in septic shock patients vs. those in non‐shock patients (*p* < 0.0001, Figure 1C), and were also higher in non‐survivors vs. those in survivors (*p* < 0.0001, Figure 1D). Furthermore, serum IL‐39 levels at admission positively associated with SOFA scores (r = 0.6631, *p* < 0.0001) and PCT (r = 0.3822, *p* = 0.0002), CRP (r = 0.3598, *p* = 0.0005), lac (r = 0.5392, *p* < 0.0001) levels, although no correlations with WBC counts or Mon counts in sepsis patients (Figure 1E).

**FIGURE 2:**
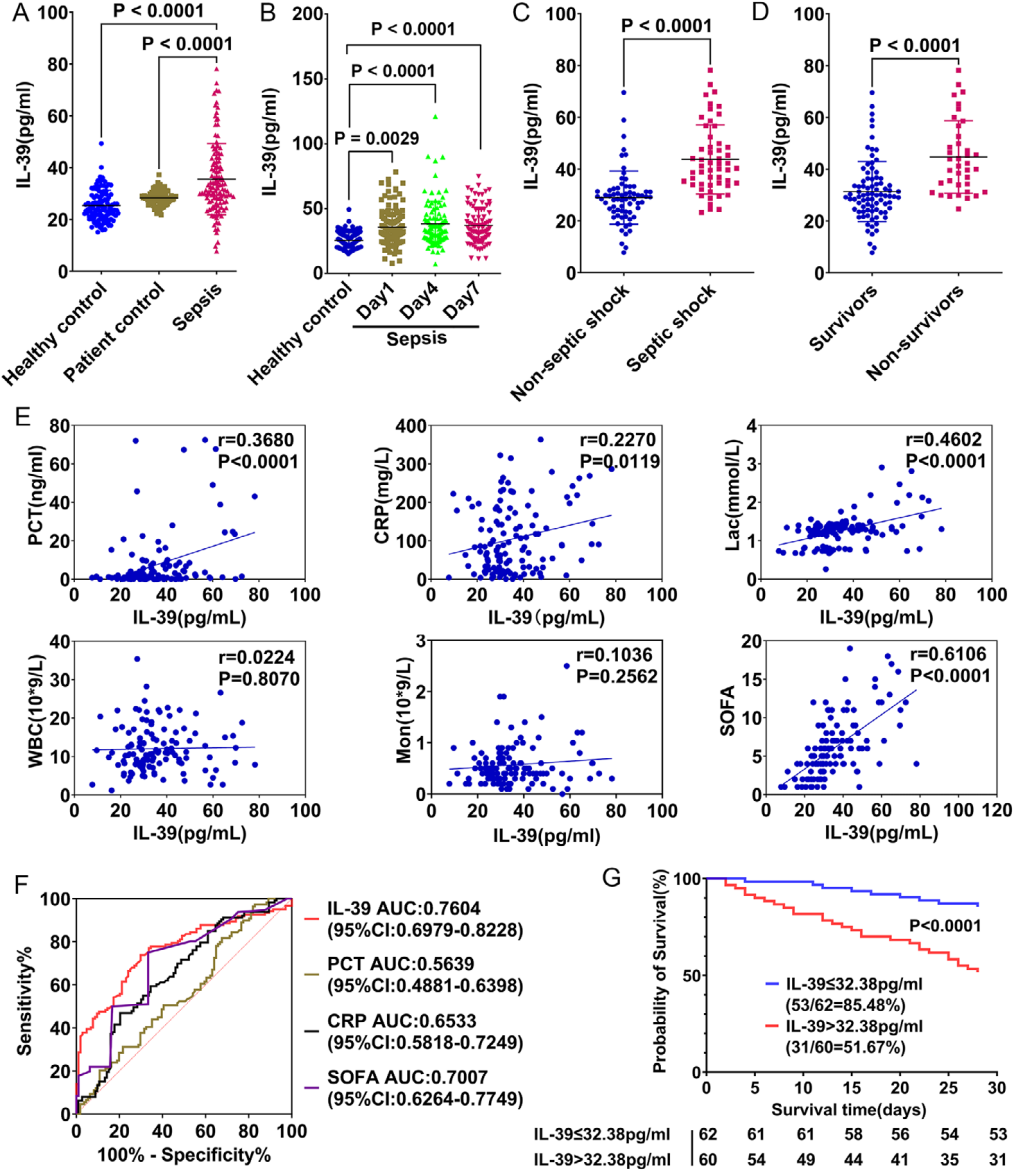
Elevated IL‐39 levels were associated with worse survival for the patients with sepsis in the Zhejiang validation cohort. (A) Levels of IL‐39 were measured by “double‐antibody sandwich” enzyme‐ linked immunosorbent assay (ELISA) in serum samples collected from healthy controls (*n* = 104), patient controls (*n* = 99) as well as patients with sepsis (*n* = 122). (B) Dynamic detection of plasma IL‐39 levels of sepsis patients at days 1, 4, and 7 compared with healthy controls. (C) Plasma levels of IL‐39 in septic shock patients (*n* = 54) and non‐shock patients (*n* = 68). (D) Plasma levels of IL‐39 in septic survivors (*n* = 84) and non‐survivors (*n* = 38). (E) Correlations between IL‐39 levels and PCT concentrations, CRP concentrations, Lau concentrations, WBC counts, Mon counts as well as SOFA scores in septic patients. (F) ROC curves for IL‐39, PCT, CRP, and SOFA at admission in predicting 28‐day mortality of patients with sepsis. (G) Kaplan‐ Meier survival curves of 122 patients with sepsis based on the IL‐39 cutoff value (32.38 pg/mL) at admission. Data are represented as mean ± SD (A–D) and determined by the Kruskal‐Wallis test followed by Dun multiple comparisons posttest in (A, B), and the two‐tailed unpaired Student's t test in (C, D). Spearman correlation coefficient (r) in (E); Kaplan‐ Meier analysis followed by log‐rank test in (F). log‐rank test in (G); ns, not significant.

The validation cohort (Zhejiang) consisted of 122 patients with sepsis, 99 patients with no‐sepsis (patient control), and 104 healthy volunteers (Table S2). The 28‐day mortality was 31.14% in Zhejiang validation patients (84 survivors, 38 non‐survivors). As observed in the Anhui cohort, serum IL‐39 levels at admission were markedly (*p* < 0.0001) higher in patients with sepsis vs. those in no‐sepsis patients or healthy controls in the Zhejiang cohort (*p* < 0.0001, Figure 2A). Consistently, serum IL‐39 in sepsis patients persisted at high levels until day 7 (*p* < 0.0001, Figure 2B), and was also increased in patients with septic shock (*p* < 0.0001, Figure 2C) or non‐ survivors (*p* < 0.0001, Figure 2D). Admission concentrations of serum IL‐39 levels were also positively associated with SOFA scores and PCT, CRP, and lac levels, but there was no correlation of IL‐39 levels with WBC counts or Mon counts in sepsis patients (Figure 2E). Together, these data suggest that elevated serum IL‐39 levels are associated to the pathogenesis of sepsis.

### 3.2 Circulating IL‐39 Levels Predict Survival in Patients With Sepsis

To evaluate the prognostic utility of serum IL‐39, we assessed the predictive accuracy for 28‐day mortality by generating receiver operating characteristic (ROC). The areas under the ROC values (AUC) for prediction of 28‐day mortality were calculated for IL‐39 levels, PCT levels, CRP levels, and SOFA scores. In the Anhui cohort, the maximal area under the curve (AUC) was identified for serum IL‐39 levels [0.7869 (95% CI: 0.7175–0.8563)], followed by SOFA scores [0.6624 (95% CI: 0.5834–0.7414)], PCT [0.5843 (95% CI: 0.4886–0.6800)] and CRP [0.5600(95% CI: 0.4752–0.6448)] (Figure 1F). Patients with higher serum IL‐39 levels had poorer survival when using a threshold of 32.38 pg/mL (Figure 1G).

We further evaluated the utility of serum IL‐39 levels at admission to predict 28‐day mortality in the Zhejiang validation cohort. Consistent with the Anhui cohort, the AUC value of IL‐39 was higher than SOFA scores, CRP levels, and PCT levels (Figure 2F). Consistently, the survival rates of patients with higher serum IL‐39 levels were also markedly poorer in the Zhejiang cohort. (Figure 2G). Collectively, these data suggested that excessive production of IL‐39 may lead to poor prognosis in sepsis.

### 3.3 Elevated IL‐39 Primarily Derived from Macrophages in Polymicrobial Sepsis

**FIGURE 3:**
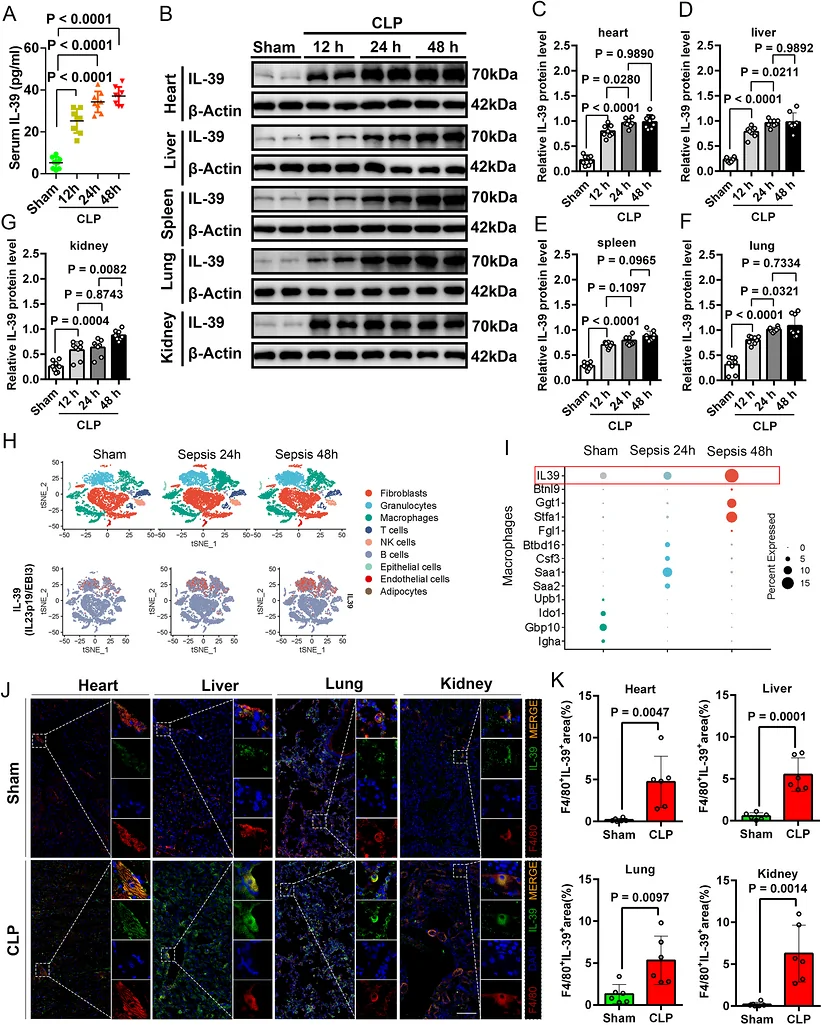
IL‐39 expression is up‐regulated in macrophages in sepsis. (A) Serum IL‐39 concentrations of septic mice at 12, 24, and 48 h after CLP were measured by “double‐antibody sandwich” ELISA (n = 8 per group). (B) The IL‐39 protein expression in heart, liver, spleen, lung, and kidney from male mice at 12, 24, and 48 h after CLP was assessed via Native‐PAGE Western blot. (C–G) The relative protein levels of IL‐39(IL23p19/EBI3) in the tissues were calculated. (H) The lung tissue obtained from septic mice at 24 or 48 hafter CLP was analyzed by scRNA‐seq. t‐Distributed stochastic neighbor embedding plot displaying the major cell types in septic mice and feature plot showing expression of IL‐39 (IL23p19/EBI3) across all populations. (I) Bubble plot showing expression of the top 13 DEGs in monocyte clusters within sepsis. (J) Immunofluorescence staining of F4/80 (red) and IL‐39(EBI3) (green) in tissues from mice with sepsis. DAPI (blue) was used to stain nuclei. (K) The fluorescence intensity of IL‐39 in macrophages was calculated. Data are represented as mean ± SD and determined by Kruskal‐Wallis test followed by Dun multiple comparisons posttest in (A, C–G), the two‐tailed unpaired Student's t test in (K).

To determine the changes of circulating IL‐39 levels during sepsis, the cecal ligation and puncture (CLP)‐induced polymicrobial sepsis model was used, and peripheral blood and tissues were collected 24 h after CLP. We observed that serum IL‐39 levels were continuously elevated after CLP (Figure 3A). In addition, the expression of IL‐39 was also increased in the heart, liver, spleen, lung, and kidney tissues of septic mice (Figure 3B–G). These results indicate that IL‐39 protein expression is upregulated in the mouse model of sepsis, similar to findings observed in septic patients.

To explore the specific source of circulating elevated IL‐39 during sepsis, we analyzed single‐cell sequencing profiles of lung tissues which are a primary vulnerable organ during sepsis progression, and sepsis‐induced acute lung injury is a dominat contributor to adverse sepsis outcomes [22]. Furthermore, previous studies have confirmed that IL‐39 is highly expressed within the lung immune microenvironment [23]. We found that IL‐39 was expressed in monocytes/macrophages, granulocyte, T cells, and et al., especially in monocytes/macrophages in septic mice (Figure 3H and Figure S1D,E). Notably, IL‐39 was significantly upregulated in macrophages (Figure 3I). With this in mind, we further determined that the expression of IL‐39 using whole tissue immunofluorescence and determined that IL‐39 was widely expressed in F4/80+ macrophages within the heart, lung, liver, and kidney tissues of septic mice (Figure 3J). Consistently, higher levels of IL‐39 fluorescence intensity were detected in F4/80+ macrophages in the heart, liver, lung, and kidney tissues of septic mice (Figure 3K). These results demonstrated that IL‐39 is up‐regulated by macrophages in response to sepsis.

### 3.4 Replenishment of IL‐39 Profoundly Exacerbates Hyper‐Inflammation and Organ Dysfunction in Polymicrobial Sepsis

To elucidate the role of IL‐39 in the pathogenesis of sepsis, we administered recombinant IL‐39 (rmIL‐39) to CLP mice. We observed that early treatment with rmIL‐39 (0.125, 0.25, or 0.5 µg/g) at the time of CLP significantly increased lethality compared with vehicle‐treated controls (Figure S2A). Consistently, delayed treatment with rmIL‐39 (0.5 µg/g) after the mice displayed clinical manifestations (at 0 or 6 or 12 hafter CLP) also increased lethality (Figure S2B). In addition, rmIL‐39 increased CLP‐induced lethality in a sex‐independent manner (Figure S2C). Moreover, in order to mimic human clinical scenarios, imipenem‐cilastatin was injected into the mice 8, 16, and 24 h after CLP, then observed that rmIL‐39‐treated mice had higher lethality in the presence of antibiotics than vehicle‐treated controls (Figure S2D).

The higher lethality of septic mice with rmIL‐39 treatment was related with increased evidence of organ injury, such as hepatocellular injury (AST, ALT), kidney injury (Cr, urea), and general cellular injury (LDH) in rmIL‐39‐treated mice after CLP (Figure S2E). Tissue injury was also analyzed by hematoxylin and eosin staining. Similarly, the incidence of myocardial edema, hepatic hemorrhage, pulmonary destruction, and interstitial edema, and observable damage to the kidney parenchyma were markedly higher in the rmIL‐39 treated septic mice than in the vehicle‐treated control mice after CLP (Figure S2F). Moreover, the incidence of inflammatory cell infiltration (neutrophils) (Figure S2G) and the expression of pro‐inflammatory factors (IL‐6, IL‐1β, and TNF‐α) were also significantly increased, while anti‐inflammatory factor (IL‐10) expression was decreased (Figure S2H), in the rmIL‐39 treated septic mice compared with that in vehicle‐treated control mice after CLP. Thus, these findings suggesting that IL‐39 had direct effects on the induction of lethality, multiple organ dysfunction, tissues damage, and inflammatory response in polymicrobial sepsis.

**FIGURE 4:**
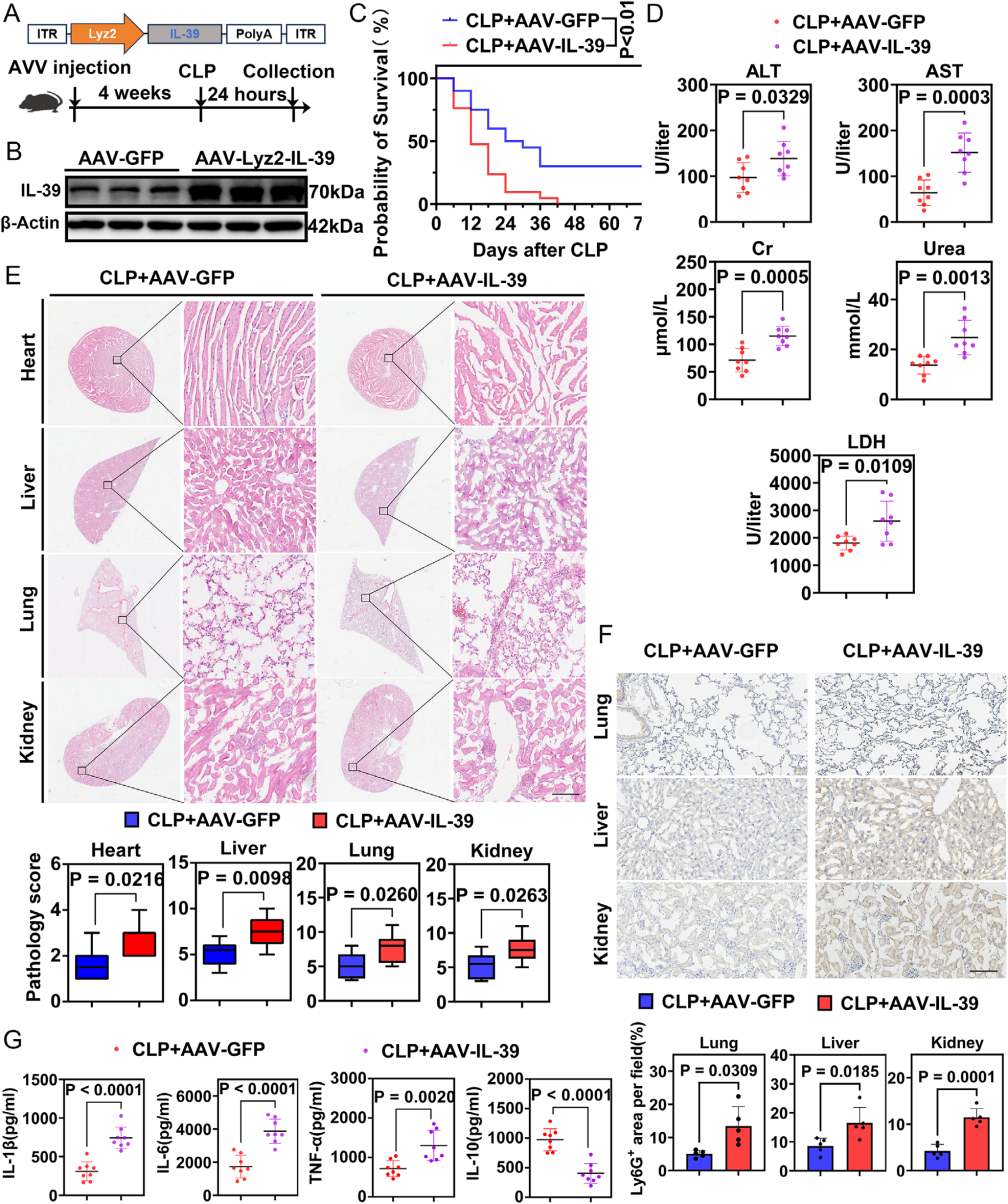
Macrophage‐specific overexpression of IL‐39 deteriorates sepsis‐induced multiple organ injury and mortality in mice. (A) Schematic diagram of AAV‐mediated macrophage‐specific overexpression of IL‐39 (IL23p19/EBI3) experiment in mice. 4‐week‐old male WT mice were treated with AAV‐Lyz2‐IL‐39(IL23p19/EBI3) or AAV‐GFP‐RNA by tail vein injection. After 4 weeks of AAV injection, all mice underwent either CLP or sham. (B) Macrophage (BMDMs) IL‐39 protein levels in mice treated with or without AAV‐Lyz2‐IL‐39, detected by Native‐PAGE Western blot (*n* = 3). (C) Survival was monitored for 72 hafter CLP (CLP+AAV‐GFP‐RNA, *n* = 20; CLP+AAV‐Lyz2‐IL‐39, *n* = 21). (D) Serological markers of organ injury, including AST, ALT, LDH, creatinine (Cr), and urea from CLP mice (*n* = 8 per group). (E) Representative examples of hematoxylin and eosin‐stained heart, liver, lung, and kidney tissues. Scale bars, 100 µm. Histological scores. (F) Neutrophil (Ly6G+) accumulation in lung, liver, kidney tissues. (G) Serological markers of pro‐inflammatory factors (IL‐6, IL‐1β, and TNF‐α) and anti‐inflammatory factor (IL‐10). Data are represented as mean ± SD and determined by the Kruskal‐Wallis test followed by the two‐tailed unpaired Student's t test in (D, F, G). Data are represented as Median (Quartile) [M (P25, P75)] (E) and analyzed using the Kruskal‐Wallis test (E). Kaplan‐Meier analysis followed by log‐rank test in (C).

Considering an obvious elevation of macrophage IL‐39 contents in CLP, we next explored the role of macrophage‐derived IL‐39 in CLP. Hence, mice with macrophage‐specific overexpression of IL‐39 were generated by intravenous (i.v.) injection of AAV9‐encoding IL‐39 under the control of the Lyz2 promoter (AAV‐IL‐39) and evaluated at protein levels then subjected to CLP (Figure 4A,B and Figure S7). Interestingly, mice treated with AAV‐IL‐39 exhibited higher lethality in comparison with AAV‐GFP‐treated control mice after CLP (Figure 4C). The higher lethality of IL‐39 overexpression was associated with increased AST, ALT, Cr, urea, and LDH in AAV‐IL‐39 treated mice 24 h after CLP (Figure 4D). Consistently, the incidence of myocardial edema, hepatic hemorrhage, pulmonary destruction, and interstitial edema, and observable damage to the kidney parenchyma was obviously higher in the AAV‐IL‐39 treated septic mice than in the AAV‐GFP‐treated control mice after CLP (Figure 4E). Moreover, neutrophils infiltration (Figure 4F) and IL‐6, IL‐1β, and TNF‐α levels were also markedly increased, while IL‐10 levels were decreased (Figure 4G), in the AAV‐IL‐39 treated mice than that in AAV‐GFP‐treated control mice after CLP. Thus, these data suggest that macrophage‐derived IL‐39 directly induces hyper‐inflammation to deteriorate the pathology of sepsis.

### 3.5 Macrophage IL‐39 Deficiency Protects Septic Mice from Lethality by Mitigating Inflammation and Organ Damage during Polymicrobial Sepsis

To further determine the role of IL‐39 in the pathogenesis of sepsis, mice with macrophage‐specific silencing of IL‐39 were generated by intravenous (i.v.) injection of AAV9‐encoding IL‐39 under the control of the Lyz2 promoter (AAV‐shIL‐39) and evaluated at protein levels in macrophages (BMDMs), then subjected to CLP (Figures S3A,B and S7). Interestingly, mice treated with AAV‐shIL‐39 exhibited lower lethality in comparison with AAV‐GFP‐treated control mice after CLP (Figure S3C). The lower lethality of IL‐39 deficiency was associated with decreased AST, ALT, Cr, urea, and LDH levels in AAV‐shIL‐39 mice 24 h after CLP (Figure S3D). Likewise, the incidence of myocardial edema, hepatic hemorrhage, pulmonary destruction, and interstitial edema, and observable damage to the kidney parenchyma were markedly lower in the AAV‐shIL‐39 treated mice compared with that in the AAV‐GFP‐treated control mice after CLP (Figure S3E). Moreover, neutrophils infiltration (Figure S3F) and IL‐6, IL‐1β, and TNF‐α levels were also markedly decreased, while IL‐10 levels were increased (Figure S3G), in the AAV‐shIL‐39 septic mice compared with AAV‐GFP‐treated control mice after CLP. Thus, these findings suggest that IL‐39 deficiency reduces lethality, multiple organ dysfunction, tissue damage, and inflammatory response in polymicrobial sepsis.

Next, we hope to determine the role of IL‐39 in the pathogenesis of sepsis through conditional knockout of IL‐39 in macrophages. IL‐39 is a heterodimer composed of the Ebi3 beta subunit and IL‐23p19 alpha subunit. Meanwhile, deficiency of a subunit leads to assembly obstacles [8, 9]. Then, macrophage‐specific Cre (Lyz2‐Cre) mice were bred with IL‐39(Ebi3)‐floxed (Ebi3^F/F^) mice to generate macrophage‐specific IL‐39^KO^ (Ebi3^MKO^) mice (Figure S4A,B). Our findings demonstrated that Ebi3 deficiency abrogates the formation of the IL‐39 heterodimer (Figure S4C). Under physiological conditions, the deficiency of IL‐39(Ebi3) does not affect organ function or morphology (Figure S4D,E). Expectedly, in line with the findings in AAV‐shIL‐39 mice, IL‐39^KO^ (Ebi3^MKO^) mice with CLP exhibited a significant decrease in mortality compared to IL‐39^WT^ (Ebi3^WT^) controls (Figure S5B). Accordingly, IL‐39^KO^ (Ebi3^MKO^) mice displayed notable mitigation of multiple organ dysfunction, tissue damage, and inflammatory response compared with the controls (Figure S5C–F). In addition, we have performed rescue experiments and further determined the pathogenic role of endogenous IL‐39 in sepsis. Specifically, AAV‐Lyz2‐IL‐39 was administered to restore IL‐39 expression in IL‐39^KO^ (Ebi3^MKO^) mice. We also observed that relative to naive IL‐39^KO^ (Ebi3^MKO^) mice, AAV‐Lyz2‐IL‐39‐treated IL‐39^KO^ (Ebi3^MKO^) mice displayed worsened survival outcomes and more severe multiple organ damage (Figure S11). These data indicated that Macrophage‐derived IL‐39 is indispensable in sepsis induced hyper‐inflammation, multiple organ dysfunction and damage, and lethality.

### 3.6 IL‐39 Targets GP130 Through the Ligand‐Receptor Pathway

**FIGURE 5:**
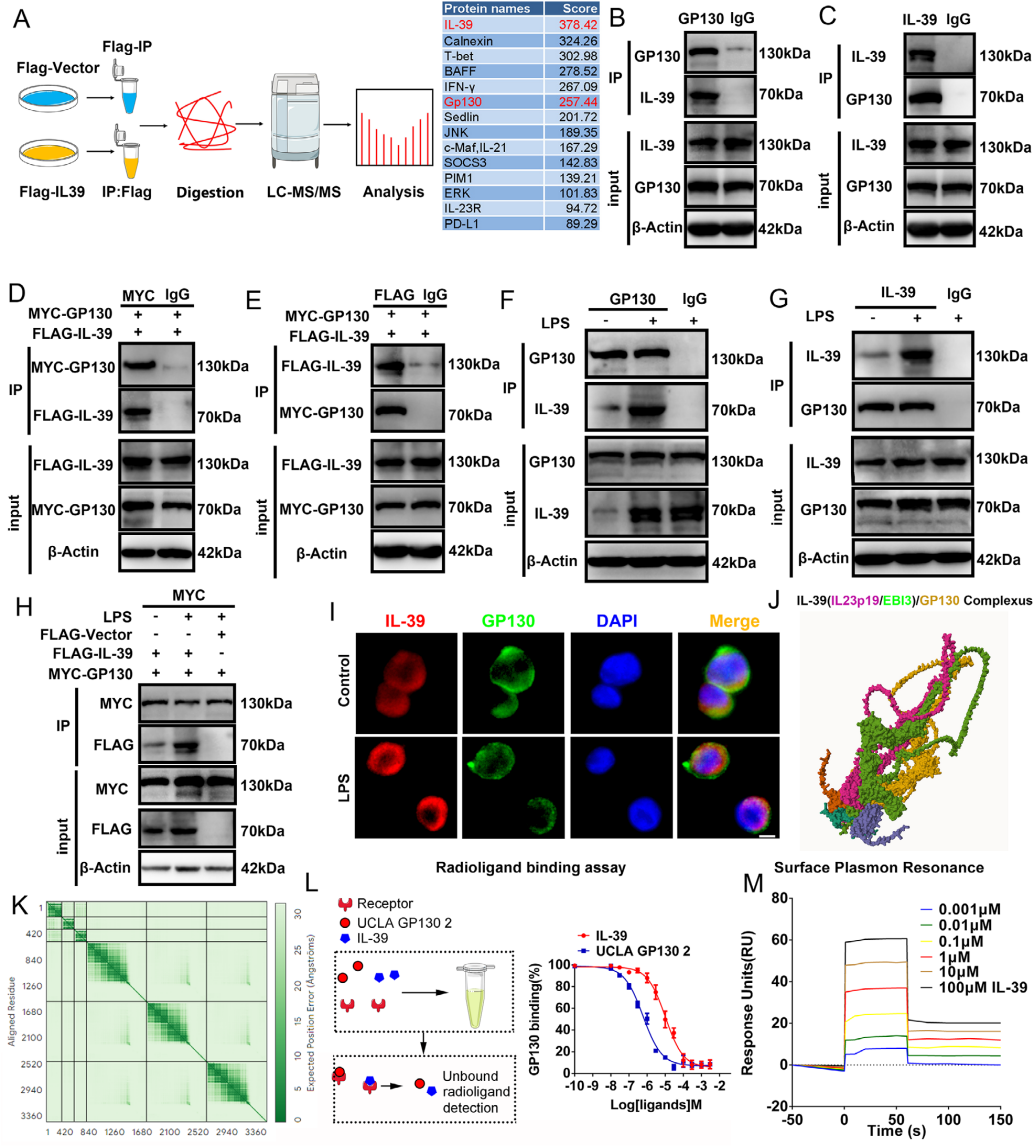
IL‐39 interacts with GP130 through the ligand‐receptor pathway. (A) IL‐39(IL23p19/EBI3)‐interacting proteins were identified by mass spectrometry. The top 14 significantly different proteins were listed. (B, C) Proteins were immunoprecipitated from whole‐cell lysates of primary macrophages with anti‐GP130 (B) or anti‐IL‐39(EBI3) (C) antibodies, followed by Native‐PAGE Western blot analysis. (D and E) RAW264.7 cells were transfected with the indicated plasmids for 24 h. Proteins were immunoprecipitated from whole‐cell lysates with anti‐MYC (D) or anti‐FLAG (E) antibodies, followed by Native‐PAGE Western blot analysis. (F–G) Primary macrophages left untreated or stimulated with LPS for 24 h. Proteins were immunoprecipitated from whole‐cell lysates with anti‐GP130 antibody (E) or anti‐IL‐39(EBI3) (G) antibodies, then analyzed by Native‐PAGE Western blot. (H) After transfection with indicated plasmids, RAW264.7 cells were left untreated or stimulated with LPS for 24 h. Proteins were immunoprecipitated from whole‐cell lysates with anti‐MYC antibody and then analyzed by Native‐PAGE Western blot. (I) Immunofluorescence staining of IL‐39(EBI3) and GP130 (green) in untreated and LPS ‐stimulated primary macrophages. (Scale bar, 10 µm.) (J) Predicting the 3‐Dimensional spatial structure of the IL39(IL23p19/EBI3)/GP130 complex using AlphaFold3.0. Green: IL39 (EB3I subunit), Red: IL39 (IL‐23p19 subunit), and Yellow: GP130. (K) The specific domain locations of the interaction between IL39(IL23p19/EBI3) with gp130 predicted by AlphaFold3.0. (L, M) Schematic diagram illustrating the radio ligand binding assay. Ligand specificity. IL‐39 (red), UCLA GP1302 (blue, GP130 agonist) interactions with GP130 were monitored using β‐arrest in system overexpressing GP130. (M) Kinetic binding analysis of IL‐39 to immobilized GP130 measured by surface plasmon resonance (SPR). Serial concentrations of recombinant IL‐39 (0.001, 0.01, 0.1, 1, 10, and 100 µm) were flowed over GP130‐coupled sensor chips. RU, Response Units.

To explore the potential downstream targets mediating the pathogenic effects of IL‐39 in the progression of sepsis, we screened for IL‐39‐interacting proteins through immunoprecipitation and mass spectrometry (Figure 5A). GP130, the receptor of EBI3 (a subunit of IL‐39) [24], was identified as a potential binding partner of IL‐39. To confirm the direct interaction between IL‐39 and GP130, we performed co‐immunoprecipitation and found that endogenous IL‐39 interacted with GP130 in primary macrophages and RAW264.7 cells (Figure 5B,C). In addition, exogenously expressed FLAG‐IL‐39 was found to interact with exogenously expressed MYC‐GP130 in transfected cells (Figure 5D,E). Moreover, LPS stimulation strengthened the interaction between IL‐39 and GP130 at both endogenous (Figure 5F,G) and exogenous levels (Figure 5H). Immunofluorescence staining also confirmed that IL‐39 colocalized with GP130 in the cytoplasm of primary macrophages (Figure 5I). Then, to investigate the binding mode underlying this potential interaction, we predicted the 3‐Dimensional spatial structure of the IL39/GP130 complex using *AlphaFold3.0* (Figure 5J) and speculated on the potential sites of the interaction between IL39 and gp130 (Figure 5K, ipTM = 0.25 pTM = 0.32). Then, we conducted whole‐cell radioligand competitive binding assays using radiolabeled IL‐39 and extracted cellular membranes transfected with GP130. The results demonstrated that IL‐39 could competitively bind to GP130 with UCLA GP130 2 (GP130 agonist), with a pKi value of 7.67 ± 1.05 nm (Figure 5L). Plasmon Resonance (SPR) assays further confirmed that IL‐39 directly targets GP130 (Figure 5M). Together, these data show that IL‐39 directly interacts with GP130 through the ligand‐receptor pathway.

### 3.7 GP130 Deficiency Abolishes IL‐39‐Triggered Exacerbations for Sepsis

**FIGURE 6:**
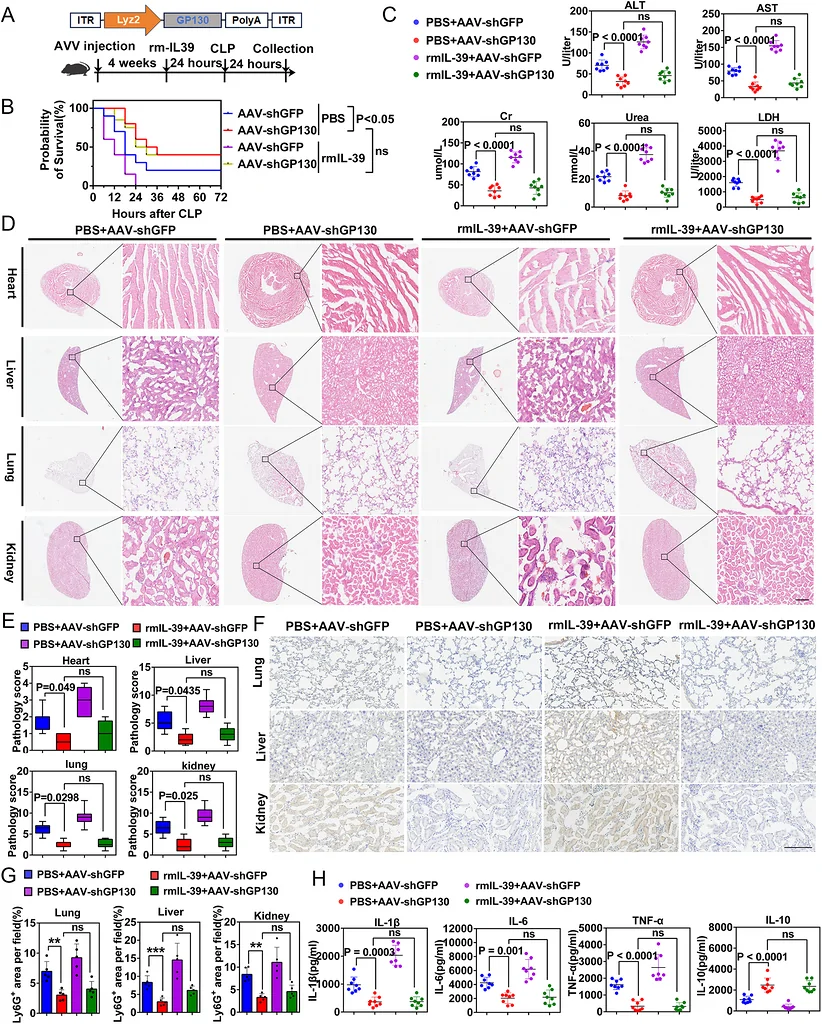
Deficiency of GP130 mitigates IL39‐induced inflammation, multiple organ injury, and mortality in septic mice. (A) Schematic diagram of AAV‐mediated macrophage‐specific silencing of GP130 experiment in mice. 4‐week‐old male WT mice were treated with AAV‐Lyz2‐sh GP130 or AAV‐GFP‐shRNA by tail vein injection. After 4 weeks of AAV injection, all mice received either rm‐IL39 or PBS. CLP or sham was performed 24 h later. (B) Survival was monitored for 72 hafter CLP (*n* = 20 per group). (C) Serological markers of organ injury, including AST, ALT, LDH, creatinine (Cr), and urea from CLP mice (*n* = 8 per group). (D, E) Representative examples of hematoxylin and eosin‐stained heart, liver, lung, and kidney tissues and histological scores. Scale bars, 100 µm. (F, G) Neutrophil (Ly6G+) accumulation in tissues. (H) Serological markers of pro‐inflammatory factors (IL‐6, IL‐1β, and TNF‐α) and anti‐inflammatory factor (IL‐10). Data are represented as mean ± SD and determined by the Kruskal‐Wallis test followed by the two‐tailed unpaired Student's t test in (C, G, H). Data are represented as Median (Quartile) [M (P25, P75)] (E) and analyzed using the Kruskal‐Wallis test. Kaplan‐ Meier analysis followed by log‐rank test in (B).

GP130 has been reported to promote inflammation and exacerbate sepsis‐induced organ dysfunction [25, 26]. To determine whether IL‐39 contributes to the progression of sepsis in dependence on GP130, mice with macrophage‐specific silencing of GP130 were generated by intravenous (i.v.) injection of AAV9‐encoding GP130 under the control of the Lyz2 promoter (AAV‐shGP130) and then subjected to CLP with recombinant IL‐39 (rmIL‐39) treatment (Figure 6A). Consistently, silencing GP130 in macrophage significantly decreases lethality (Figure 6B), multiple organ dysfunction (Figure 6C), tissues damage (Figure 6D,E), neutrophil infiltration (Figure 6F,G), and Inflammatory factor expression (Figure 6H) in septic mice. As expected, rmIL‐39 treatment‐induced effects to sepsis progression were significantly reversed in AAV‐shGP130‐treated mice (Figure 6). To further confirm the role of GP130 as a downstream target of IL‐39 mediating its pathogenic role in sepsis, AAV‐Lyz2‐shGP130 was injected into macrophage‐specific IL‐39^KO^ (Ebi3^MKO^) mice or littermates wild‐type (Ebi3^WT^) mice. Expectedly, in line with the findings in rmIL‐39 treatment mice, silencing of macrophage GP130 by AAV‐Lyz2‐sh GP130 eliminated the protection of IL‐39 deficiency (Ebi3^MKO^) against sepsis (Figure S6). Collectively, these results demonstrate that GP130 is a downstream target that mediates IL‐39‐triggered exacerbations in the pathological progression of sepsis.

### 3.8 IL‐39 Targets Gp130 to Promotes Mitogen‐Activated Protein Kinase (Mapk) Phosphorylation/P38 Nuclear Translocation and Subsequently Il‐6, Il‐1β, and Tnf‐Α Generation

**FIGURE 7:**
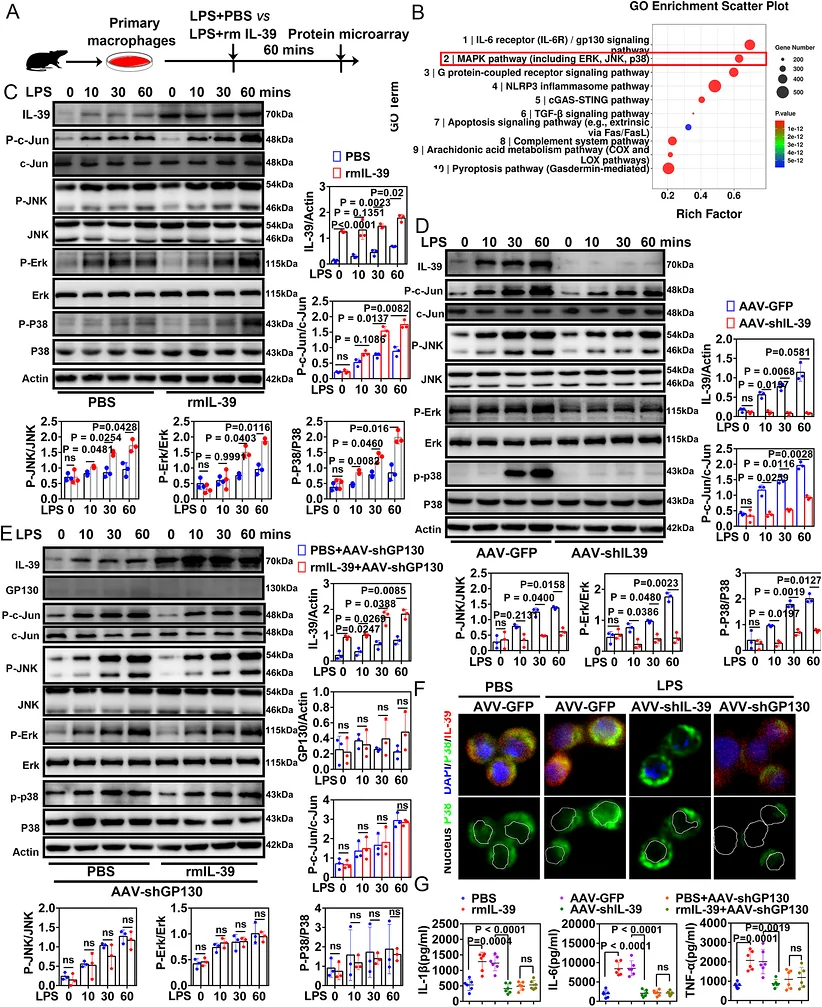
IL‐39 targets GP130 to promote mitogen‐activated protein kinase (MAPK) phosphorylation and P38 nuclear translocation and subsequently inflammation. (A) The study protocol is shown. rmIL‐39‐ or vehicle‐treated LPS‐stimulated primary macrophages were analyzed by proteomics using a protein microarray (*n* = 3). (B) Top 10 pathway enrichment of proteomics in rmIL‐39‐ or vehicle‐ treated LPS‐stimulated primary macrophages using GO analysis. the gp130 signaling pathway and MAPK pathway (including Erk, JNK, and p38) are highlighted in red. (C) Primary macrophages were treated with LPS (100 ng/mL) for 0, 10, 30, and 60 min, then total cellular proteins were extracted to detect the expression of IL‐39 and the activation of MAPK (including phospho‐c‐Jun, phospho‐JNK, phospho‐Erk, and phospho‐p‐38) by Western blot analysis (*n* = 6). (D) 4‐week‐old male WT mice were treated with AAV‐Lyz2‐sh IL‐39(IL23p19/EBI3) or AAV‐GFP‐shRNA by tail vein injection. After 4 weeks of AAV injection, primary macrophages were extracted and treated with LPS (100 ng/mL) with 0, 10, 30, and 60 min, then the expression of IL‐39 and the activation of MAPK were detected by Western blot analysis. (E) 4‐week‐old male WT mice were treated with AAV‐Lyz2‐shGP130 or AAV‐GFP‐shRNA by tail vein injection. After 4 weeks of AAV injection, primary macrophages were extracted and treated with LPS+rmIL‐39 (10 ng/mL) or LPS + PBS, then the expression of IL‐39, GP130, and the activation of MAPK were detected by Western blot analysis. (F) The nuclear translocation of p38 in macrophages were detected by Immunofluorescence. (G) Pro‐inflammatory factors (IL‐6, IL‐1β, and TNF‐α) in macrophages lysates were detected by ELISA. Data are represented as mean ± SD and determined by Kruskal‐Wallis test followed by the two‐tailed unpaired Student's t test in.

Having demonstrated that IL‐39 through GP130 to deteriorate the pathological process of experimental sepsis, we next performed proteomics to determine downstream signaling (Figure 7A). IL‐39 triggered the activation of MAPK signaling cascades within macrophages (Figure 7B). To elucidate the intracellular molecular mechanisms in macrophages triggered by IL‐39, we examined the phosphorylation of MAPK signaling components. rmIL‐39 induced the phosphorylation of MAPK in a time‐ dependent manner of c‐ Jun N‐ terminal kinase (c‐jun/JNK), extracellular signal–regulated kinase(ERK), p38 mitogen‐activated protein kinase (p38MAPK) (Figure 7C), and P38 nuclear translocation (Figure 7F) and subsequently IL‐6, IL‐1β, and TNF‐α transcription and generation (Figure 7G). Consistently, macrophage‐specific IL‐39 loss inhibited the phosphorylation of MAPK, P38 nuclear translocation (Figure 7F), and subsequently IL‐6, IL‐1β, and TNF‐α generation (Figure 7G). As expected, macrophage‐specific GP130 loss suppressed IL‐39‐induced‐MAPK phosphorylation (Figure 7E), P38 nuclear translocation (Figure 7F) and subsequently IL‐6, IL‐1β, and TNF‐α generation (Figure 7G). In addition, a p38‐specific inhibitor abolished IL‐39‐mediated inflammation (Figure S8). Collectively, our results suggest that macrophage IL‐39 targets GP130, promoting MAPK phosphorylation, which in turn triggers inflammation and exacerbates sepsis.

### 3.9 Pharmaceutical Inhibition of IL‐39 is a Potential Treatment for Sepsis

**FIGURE 8:**
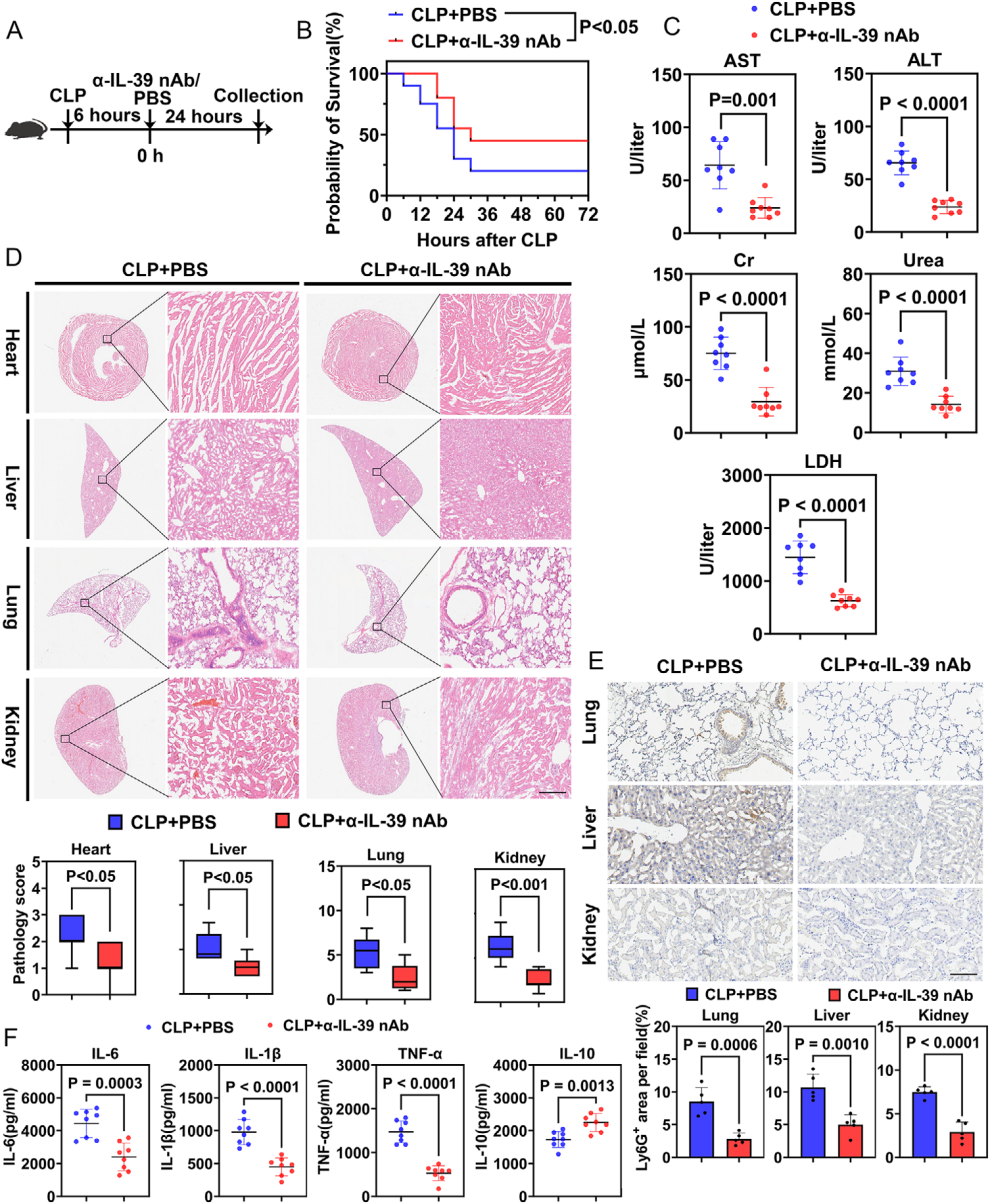
Protection from lethal sepsis by neutralizing the biological function of IL‐39. (A) anti‐IL39 neutralizing antibody (α‐IL‐39 nAb) or PBS was injected intravenously (i.v.) into male mice. 24 h later, CLP was performed. (B) Survival was monitored for 72 h after CLP (*n* = 20 per group). (C) Serological markers of organ injury, including AST, ALT, LDH, creatinine (Cr), and urea from CLP mice (*n* = 8 per group). (D) Representative examples of hematoxylin and eosin‐stained heart, liver, lung, and kidney tissues. Scale bars, 100 µm. Histological scores. (E) Neutrophil (Ly6G+) accumulation in lung, liver, kidney tissues. (F) Serological markers of pro‐inflammatory factors (IL‐6, IL‐1β, and TNF‐α) and anti‐inflammatory factor (IL‐10). Data are represented as mean ± SD and determined by the Kruskal‐Wallis test followed by the two‐tailed unpaired Student's t test in (C, D, E). Data are represented as Median (Quartile) [M (P25, P75)] (D) and analyzed by using the Kruskal‐Wallis test. Kaplan‐Meier analysis followed by the log‐rank test in (B).

To further investigate the potential applications of IL‐39, we investigated whether blocking IL‐39 with neutralizing antibodies can protect against sepsis‐induced organ dysfunction and damage. We observed that treatment with anti‐IL‐39 neutralizing antibody significantly reduced lethality (Figure 8B), multiple organ dysfunction (Figure 8C), tissue damage (Figure 8D), neutrophil infiltration (Figure 8E), and inflammatory factor expression (Figure 8F), and increased the anti‐inflammatory factor (IL‐10) expression (Figure 8F) in septic mice. Meanwhile, anti‐IL‐39 neutralizing antibody inhibited the activation of MAPK and the subsequent production of inflammatory factors (Figure S9). Furthermore, anti‐IL‐39 neutralizing antibody‐treatment mice displayed lower bacterial loads in the blood and peritoneal lavage fluid (PLF) and enhanced bacterial phagocytosis (Figure S10). These results suggest that pharmaceutical inhibition of IL‐39 by neutralizing antibody is a potential treatment strategy for sepsis.

## 4 Discussion

Here, we propose the novel cytokine IL‐39 as a potential prognostic biomarker and host‐directed therapy target for sepsis. IL‐39 levels were notably elevated in patients with sepsis, and elevated circulating concentrations of IL‐39 were correlated with increased mortality rates and unfavorable clinical outcomes. Elevated IL‐39 derived from macrophages and profoundly exacerbates hyper‐inflammation and organ dysfunction and damage in polymicrobial sepsis. Conversely, genetic ablation or pharmaceutical inhibition of IL‐39 significantly protects against sepsis‐induced organ dysfunction and damage. Mechanistically, IL‐39 targets GP130 through the ligand‐receptor pathway to promote MAPK phosphorylation and P38 nuclear translocation, and subsequently transcription and generation of pro‐inflammatory factors (IL‐6, IL‐1β, and TNF‐α). Collectively, these results elucidate the detrimental role of IL‐39 in sepsis pathophysiology as a disease‐promoting immune checkpoint that can be inhibited to protect against sepsis.

Effectively and precisely determining the immune status of patients with sepsis is crucial for targeted therapy based on specific pathophysiological factors [27, 28, 29]. Recently, the role of novel immunomodulatory factors such as IL‐17D, IL‐36, and IL‐40 in regulating overactive inflammatory immune responses for sepsis has attracted widespread attention [3, 4, 5, 30]. These immunomodulatory proteins have shown high clinical practical value in the early diagnosis and therapeutic intervention of sepsis, and have been recognized as candidate biomarkers for sepsis diagnosis and prognosis [31, 32, 33].

In this work, we report a novel cytokine IL‐39 is a new candidate biomarker for sepsis diagnosis and treatment. We measured the serum levels of IL‐39 in sepsis patients in two independent cohorts of patients with sepsis and found that circulating IL‐39 levels of sepsis patients on the day of admission were significantly higher compared with those of ICU controls and healthy individuals. Moreover, high circulating IL‐39 levels were related to elevated PCT, CRP, Lau levels, and higher SOFA scores. Importantly, elevated circulating IL‐39 levels at admission had higher risk of 28‐day mortality in septic patients. Furthermore, as a small secretory protein, IL‐39 protein in serum can be easily measured using existing automated testing methods or manual ELISA methods in routine and emergency laboratories of the clinical laboratory [13, 14, 16, 18]. In addition, IL‐39 has good stability, and its elevated expression in peripheral circulation can last up to 7 days after admission, which helps to monitor the condition and prognosis for patients with sepsis.

Early excessive inflammation inevitably leads to later immunosuppression, which is a key characteristic of sepsis. Therefore, avoiding an excessive inflammatory response is crucial for the treatment of sepsis [1]. This study demonstrates that IL‐39 plays a pathogenic role in sepsis by promoting the generation of pro‐inflammatory factors, which recruit neutrophils and subsequently expand the “inflammatory storm” to deteriorate sepsis. Moreover, to evaluate the potential applications of IL‐39, we used neutralizing antibodies and adenovirus gene therapy to block IL‐39 and determined that blocking IL‐39 significantly reduced the secretion of macrophage inflammatory factors and the resulting organ damage and dysfunction, suggesting that both endogenous and secreted IL‐39 can activate a pro‐inflammatory sepsis phenotype, and anti‐IL‐39 therapy including neutralizing antibodies or gene therapy may constitute a novel therapeutic strategy for sepsis.

In further investigations, by using mass spectrometry and proteomics, we determined that the role of IL‐39 in promoting Inflammatory response and sepsis progression was highly related to its interaction with GP130 in macrophages. GP130 serves as the ubiquitous signal‐transducing receptor subunit for the IL‐6 cytokine family, which includes complex cytokines containing the EBI3 subunit, such as IL‐39 [24, 34]. The biological pleiotropy of GP130 is fundamentally determined by its activation mode: it is either recruited via the binding of cytokines to membrane‐bound receptors (classic signaling) or activated through soluble receptor‐cytokine complexes (trans‐signaling) [24, 34]. We demonstrated a robust interaction between IL‐39 and GP130 in macrophages, and subsequent functional assays revealed that IL‐39 activates GP130 through a canonical ligand‐receptor mechanism (classic signaling). Several studies established that GP130 exacerbates sepsis pathogenesis through multiple mechanisms, such as inducing the degradation or remodeling of endothelial tight junction proteins, promoting the expression of tissue factor in endothelial cells to trigger thrombosis, and stimulating the release of chemokines to recruit neutrophils [25, 35]. In contrast, evidence also suggests that GP130 acts as a protective role for immune homeostasis during sepsis. Specifically, GP130 signaling in myeloid compartments is indispensable for protective anti‐inflammatory responses and preventing unrestrained systemic inflammation [36]. Our study demonstrates that macrophage GP130 signaling drives the expression of pro‐inflammatory factors, thereby deteriorates multi‐tissue damage, organ dysfunction, and lethality in experimental sepsis. Our and previous research suggest that the dichotomous pro‐ and anti‐inflammatory roles of GP130 are highly context‐dependent, governed by the pathological stage of sepsis, host genotype, and tissue‐specific cellular environments.

Our study has several limitations. First, the relatively modest sample size limited the statistical power of the results, resulting in only moderate predictive accuracy of IL‐39 for sepsis prognosis. Larger prospective clinical trials are warranted to validate these preliminary findings. Furthermore, the potential crosstalk between the IL‐39/GP130 axis in other immune cell subsets and the relationship of IL‐39 with other interacting proteins (Calnexin et al.) during sepsis remains to be fully elucidated.

In short, our study demonstrates that sepsis triggers a significant increase in IL‐39 production, with circulating levels markedly elevated and positively correlated with disease severity and poor clinical prognosis of sepsis. Mechanistically, macrophage‐derived IL‐39 activates the GP130 signaling pathway via a ligand‐receptor interaction, thereby fueling the pro‐inflammatory expression that exacerbates hyper‐inflammation, organ dysfunction, and tissue damage in polymicrobial sepsis. These findings position IL‐39 as a promising prognostic biomarker and a viable therapeutic target for improving sepsis outcomes.

## Author Contributions

P.H.Z. and S.W.J conceived the project and designed experiments; F.Z.Z. and H.J.L. conceived the experiments and analyzed data; F.Z.Z., Y.Y, W.W.Z., J.Z., and J.J.G. performed out human sample experiments; F.Z.Z. and H.J.L. conducted the mouse experiments; Y.Y. and Y.L.Z. performed the human sample collection experiments; F.Z.Z and P.H.Z. interpreted the data and wrote the manuscript; P.H.Z., S.W.J and W.H.L. assisted in data interpretation and edited the manuscript. All authors have approved the final version of the manuscript and have agreed to be accountable for all aspects of the work.

## Funding

This study was supported by the Natural Science Foundation of Anhui Province for Excellent Young Scholars (2408085Y04), the Natural Science Foundation of Anhui Education Department (2024AH040245, 2025AHGXZK31079), the Health research project of Anhui Province (AHWJ2023A20551), the National Natural Science Foundation of China (No. 82202378, 82270098), National Natural Science Foundation of China Joint Fund Project (U22A20273), Zhejiang Province's “Jianbing Leading Goose +X” R & D Plan (2024C03188), the Talent Introduction Science Foundation of Yijishan Hospital, Young and middle‐aged research Foundation of Wannan Medical College (2023272), Anhui Province Clinical Medical Research and Transformation Special Project (202304295107020001).

## Conflicts of Interest

The authors declare no conflicts of interest.

## Supporting information




**Supporting File**: advs77663‐sup‐0001‐SuppMat.docx.

## Data Availability

The data that support the findings of this study are available from the corresponding author upon reasonable request.
