# Supplementary material for: Interleukin‐39 is a Prognostic Biomarker and Therapy Target for Sepsis
Source: Adv Sci (Weinh). 2026 Sep 10:e77663. Online ahead of print. doi: 10.1002/advs.77663 (PMC13562788; doi:10.1002/advs.77663)
Supplement: Supplementary file 1 [file ADVS-9999-e77663-s001.docx]

**Interleukin-39 is a prognostic biomarker and therapy target for sepsis**

Running title: Interleukin-39 inhibition protect against sepsis

Feng-zhi Zhang, Hai-jun Liu, Yan Yue, Jun-jie Gao, Jin Zhang, Wen-wu Zhang, Yun-long Zhang, Wei-hua Lu, Sheng-wei Jin, Pu-hong Zhang

**Contents for Supplementary Material:**

1. Supplementary Materials and Methods

2. Supplementary figures

3. Supplementary tables

**1. Supplementary Materials and Methods**

**Human study**

The discovery cohort (Anhui) comprised 155 patients (90 with sepsis and 65 with no-sepsis) admitted to the comprehensive intensive care unit (ICU) and 30 healthy age- and sex-matched volunteers admitted to the Physical Examination Center of the first Affiliated Hospital of Wannan Medical University from September 2023 to July 2024.

All sepsis patients met the requirements according to the clinical criteria of Sepsis-3 [1].To further validate the specificity of our findings, we included non-septic critically ill controls (no-sepsis patients) undergoing mechanical ventilation for non-infectious conditions, such as traumatic brain injury or abdominal surgery, consistent with our previously established recruitment protocols [2]. Clinical and demographic characteristics of all patients were recorded, including age, gender, smoking history, drinking history, complication, co-morbidities (hypertension, diabetes mellitus, chronic cardiovascular disease, cerebrovascular disease, peripheral vascular disease, chronic pulmonary disease, chronic liver disease, chronic kidney disease, malignant solid tumors) and GCS scores, SOFA scores, APACHE Ⅱ scores on admission. During the follow-up, other clinical and biological variables were collected, including type of pneumonia (CAP/HAP), pathogenic bacteria (Gram-positive/ Gram-negative/Fungus/Miscellaneous), drug (Ulinastatin, Heparin Sodium, Low Molecular Weight Heparin), invasive therapy (CRRT, Vasopressors, Blood transfusion, ECMO) and laboratory indicators (WBC, NEU, IL-6, CRP, PCT, INR, Lac) and mortality during the 28-day study period after the onset of sepsis. In accordance with the Sepsis-3 definition, septic shock was characterized by the presence of sepsis combined with refractory hypotension requiring necessitated vasopressor support to sustain a mean arterial pressure (MAP)≥65mm mmHg and was accompanied by serum lactate levels exceeding 2 mmol/L, persisting despite adequate fluid resuscitation [1a]. Patients who were minors, breast-feeding, HIV infection, organ transplantation, autoimmune diseases and under immunosuppressive therapy were excluded from the cohort study [3]. After admission, the peripheral blood samples were collected for a consecutive 7-day routine blood test, then the samples were isolated and stored at −80°C for IL-39 test. Specifically, during the first 24-hour window, baseline clinical profiles and GCS scores, SOFA scores, APACHE Ⅱ scores were established. Routine laboratory markers, such as leukocyte profiles (WBC/monocyte counts) and biochemical indicators (CRP, PCT and Lac), were analyzed in the department of Clinical laboratory. Plasma concentrations of IL-39 were detected by “double-antibody sandwich” ELISA according to manufacturer’s instructions (MyBioSource, no.8808094). This protocol was approved by the Medical Ethics Committee of the First Affiliated Hospital of Wannan Medical University (2023-S-253). As blood samples were patients’ routine blood sampling after completion of routine follow-up tests in clinic, so no informed consent was needed. Nevertheless, non-opposition to inclusion in the protocol was recorded from every patient or kinsfolk (Table S1).

The validation cohort (Zhejiang) comprised 221 patients (122 with sepsis and 99 with no-sepsis) admitted to the comprehensive intensive care unit (ICU) and 104 healthy age- and sex-matched volunteers admitted to the Clinical Pharmacy Research Center of the Second Affiliated Hospital of Wenzhou Medical University from August 2024 to July 2025. All sepsis patients met the requirements according to the clinical criteria of Sepsis-3 [1a]. Non-septic critically ill controls (no-sepsis patients) undergoing mechanical ventilation for non-infectious conditions, such as traumatic brain injury or abdominal surgery, were included [2]. Clinical and demographic characteristics of all patients were recorded, including age, gender, smoking history, drinking history, complication, co-morbidities (hypertension, diabetes mellitus, et al) and GCS scores, SOFA scores, APACHE Ⅱ scores on admission. During the follow-up, other clinical and biological variables were also collected, including type of pneumonia, pathogenic bacteria, drug, invasive therapy and laboratory indicators and mortality during the 28-day study period after the onset of sepsis. The diagnosis of septic shock is made in accordance with Sepsis-3 definition [1a]. Patients who were minors, breast-feeding, HIV infection, organ transplantation, autoimmune diseases and under immunosuppressive medication were also excluded from the cohort study [3]. The peripheral blood samples were collected for a consecutive 7-day after admission, and then were isolated and stored at −80°C for next IL-39 test. Plasma concentrations of IL-39 were detected by “double-antibody sandwich” ELISA according to manufacturer’s instructions (MyBioSource, no.8808094). This protocol was approved by the Medical Ethics Committee of the Second Affiliated Hospital of Wenzhou Medical University (2022-K-172-01). The samples were obtained from leftover serum following the completion of routine clinical laboratory testing and all data were analyzed anonymously (Table S2).

**Animals**

Specific pathogen-free, C57BL/6 wild-type mice (6-8 weeks old, 22-25g) were purchased from Jiangsu Qinglongshan Biotechnology Co., Ltd. Ebi3^flox/flox^ mice were generated using the Clustered Regularly Interspaced Short Palindromic Repeats and CRISPR-associated protein 9 (CRISPR/Cas9) system by the GemPharmatech. Two loxP sequences were inserted in the introns flanked with the exon 2-5 of Ebi3 as described in Fig. S4A. The C57BL/6 background Lyz2-Cre mice were granted by Yongliang Lou (Wenzhou Medical University School of Laboratory Medicine). To specific deletion of Ebi3 in macrophages, Lyz2-Cre^+^-Ebi3^flox/flox^ mice were generated by crossing the Lyz2-Cre mice with Ebi3^flox/flox^ mice and their littermates Lyz2-Cre^-^ -ALK1^flox/flox^ were used as controls. The deletion of Ebi3 was confirmed in offspring by genotyping tail tissue DNA with specific primer sets during polymerase chain reaction (PCR). Ebi3: 5'-CACCCTTGAACTTAGACATTTGTGG-3' (forward), 5'- AAGAACTCCCTAGACTACCCACACC-3' (reverse); Lyz2-cre: 5'- GCACACAGCTCAAATGTAGGAAACC-3' (forward), 5'- ACACAGCATTGGAGTCAGAAGGG-3'(reverse); The PCR amplification protocol for Ebi3 genotyping was performed as follows: an initial denaturation at 95°C for 5 min, followed by a touchdown phase of 35 cycles (denaturation at 95°C for 30 s, annealing starting at 60°C for 30 s with a 0.5°C decrease per cycle, and extension at 72°C for 50 s). This was followed by an additional 20 cycles of 95°C for 30 s, 60°C for 30 s, and 72°C for 50 s. The procedure concluded with a final extension at 72°C for 5 min. Lyz2-Cre genotyping of PCR was performed as follows: an initial denaturation at 95°C for 3 min, followed by 35 cycles consisting of denaturation at 95°C for 10 s, annealing at 60°C for 15 s, and extension at 72°C for 35 s. The procedure was concluded with a final extension step at 72°C for 5 min.

**Septic models and Treatment regimen**

Polymicrobial sepsis model induced by Cecum Ligation and Puncture (CLP) was established as described previously [4]. In brief, female or male C57BL/6 mice aged 6 to 8 weeks were maintained in specific pathogen-free (SPF) environments, with their well-being monitored daily by animal facility personnel. For CLP experiment, mice were initially anesthetized with a ketamine/xylazine cocktail (100/10 mg/kg, i.p.). Following abdominal disinfection, a midline laparotomy was performed to expose the cecum. Then, a penetrating puncture with a 20-gauge needle distal to the ligation site, a small droplet of feces was expressed. The cecum was then repositioned within the abdominal cavity, followed by a two-layer closure of the incision. For hemodynamic support and thermal regulation, mice were administered subcutaneous saline (50 ml/kg) and placed on a thermostatic warming station. The sham group was subjected to the same surgical exposure excluding the ligation and puncture steps. Analgesic coverage and volume expansion were maintained at 6–8 h intervals for 2 days post-surgery, and monitoring was de-escalated to every 6 hours until the end of the experiments. Only for experiments in Fig. S2D, antibiotic therapy was administered via intraperitoneal injection (Imipenem-cilastatin, 25 mg/kg, Merck & Co., Inc). The regimen was initiated at 8, 16, and 24 hours post-CLP. Postoperatively, mice were monitored for 72 hours’ survival. Recombinant murine IL-39 (DTD0312, Detai Bioengineering Co., Ltd.) was injected *i.v.* into mice at 12.5–500 μg/kg at the indicated times after CLP, and PBS was delivered in a similar fashion as vehicle control. IL-39 blockade was performed by i.v. administration of 5mg/kg of mouse anti-IL-39 antibody (α-IL-39 nAb) 24 hours before CLP. Mouse IgG2b Isotype Control (MAB004, R&D Systems) was used as isotype control.

**Quantification of human/mouse serum IL-39(IL23p19/EBI3) levels**

To specifically quantify the IL-39(IL23p19/EBI3) heterodimer, we employed a double-antibody sandwich ELISA. In this configuration, a monoclonal antibody targeting the IL23p19 subunit was used for capture, while a biotin-conjugated antibody specific for the EBI3 subunit served as the detection reagent. This dual-antibody strategy ensures the exclusive measurement of the intact IL23p19/EBI3 heterodimer, effectively preventing cross-reactivity with individual subunits. Serum concentrations were determined using commercial kits according to the manufacturers' protocols: human IL-39 was quantified via the ELISA kit (8808094, MyBioSource), and mouse IL-39 was measured using the ELISA kit (Y-EM228775, Byabscience).

**Construction of AAV-Lyz2-shIL-39(IL23p19/EBI3) for Macrophage-Specific IL-39 silence**

The AAV plasmids pAAV-Lyz2-miR30-shIL-39 and the control virus were custom-produced by Cyagen Biosciences, Inc. (Guangdong, China). IL-39 is a heterodimer cytokine composed of the IL-23p19 subunit and the EBI3 subunit [5]. To achieve macrophage-specific silencing of IL-39, a recombinant AAV vector was constructed by incorporating a shRNA sequence targeting the IL-23p19 or EBI3 subunit. shRNA-IL23p19:CAGCCAGTTCTGCTTGCAAAGCTCGAGCTTTGCAAGCAGAACTGGCTG-TTTTTT. shRNA-Ebi3:ACGGTGCCCTACATGCTAAATCTCGAGATTTAGCATGTAGG

GCACCGT-TTTTTT. The DNA fragment encoding the specific shRNA was subcloned into a pAAV-ITR-Lyz2-3×Flag plasmid, where expression was driven by the myeloid-specific Lyz2 promoter. A non-targeting scramble shRNA served as the negative control. Recombinant AAV9 vectors were produced in HEK293 cells using a standard triple-transfection protocol, involving the transgene plasmid, the p1RepCap (AAV9 chimeric) helper, and the pAdeno adenoviral helper. Following cell lysis, crude viral particles were harvested and purified via double cesium chloride (CsCl) density gradient ultracentrifugation. Viral titers were determined by RT-qPCR or dot blot hybridization. The purified stocks were reconstituted in HN buffer (pH 7.4) and stored at -80°C.For in vivo delivery, 6–8-week-old male C57BL/6J mice received systemic administration of AAV9-Lyz2-shIL-39 or AAV-shControl via tail vein injection. Due to the Lyz2 promoter, gene silencing was restricted to the myeloid compartment. Silencing efficiency was validated 4 weeks’ post-injection by measuring IL-39(IL23p19/EBI3) mRNA and protein levels in BMDMs using RT-qPCR and immunoblotting, respectively.

**Construction of AAV-Lyz2-IL-39(IL23p19/EBI3) for Macrophage-Specific IL-39 overexpress**

Recombinant adeno-associated virus 9 (AAV9) expressing IL-39(IL23p19/EBI3) was constructed by Guangdong Cyagen Biosciences, Inc. IL23p19/EBI3 was amplified by polymerase chain reaction (PCR) using the primers: IL23p19: forward 5′-GCCACC-ATGCTGGATTGCAGAGCAGTA-3' and reverse 5'-TTAAGCTGTTGGCACTAAGGG

CTC-3'. EBI3: forward 5′-GCCACCATGTCCAAGCTGCTCTTC-3' and reverse 5'-TCAGGGCTTATGGGGTGC-3'. The DNA segment was then inserted into the pAAV-ITR- Lyz2-promoter-3×Flag plasmid to construct the pAAV-ITR-Lyz2-mIL23p19-3×Flag vector or the pAAV-ITR-Lyz2-mEBI3-3×Flag vector. Empty plasmids were used as control vectors. Viral aliquots were stored at -80 °C before tail intravenous injection. Four- week-old male mice were injected with AAV at a density of 1×10^11^ viral genomes/mouse for Macrophage-specific IL-39 overexpress.

**Histopathology**

To systematically evaluate systemic organ damage, mice subjected to either sham surgery or CLP were euthanized at designated post-operative time points. Subsequently, the heart, liver, lung, kidney was harvested, fixed in buffered formalin, and processed for Hematoxylin and Eosin (H&E) staining. To ensure objectivity, histopathological assessment was performed in a blinded manner by a senior pathologist who remained unaware of the experimental groupings. For the comprehensive lung pathology assessment, several key parameters across the entire pulmonary surface were scrutinized, including bronchitis, edema, interstitial and intra-alveolar inflammation, pleuritis, endotheliitis, and the percentage of lung area exhibiting confluent inflammatory infiltrates. Each parameter was quantified using a semi-quantitative scale ranging from 0 (absent) to 4 (severe). Furthermore, the liver, and kidney were graded based on the prevalence of thrombotic events, the frequency of (micro)abscesses, the intensity of inflammatory cell infiltration, and the extent of tissue necrosis. These organs were scored on a scale of 0 (normal) to 3 (marked). The cumulative pathological score for each organ was calculated by summing the individual values of all evaluated parameters, providing a holistic measure of sepsis-induced tissue injury.

**Western blot analysis**

Mouse heart, lung, liver, and kidney tissues, as well as macrophages, were lysed in lysis buffer (RIPA: PMSF = 50:1) and then sonicated. The lysate was centrifuged at 12,000 x g for 30 minutes at 4°C, and the supernatant was collected as total cardiac protein. All operations were performed on ice. Protein concentration was determined using a BCA kit, and equimolar solutions of 30 μg/10 μl were prepared using double-distilled water and 5× loading buffer. Protein samples were separated according to molecular weight by 10% sodium dodecyl sulfate polyacrylamide gel electrophoresis at 80–120 V, and then transferred to a polyvinylidene fluoride membrane. After blocking with 10% skim milk powder for 2 hours, the membrane was washed three times with TBST for 10 minutes each time. The membrane was then incubated overnight at 4°C with the following primary antibodies: GP130(ab283685, Abcam, 1:1000), C-JUN (9165, CST, 1:1000), P-C-JUN (3270, CST, 1:1000), JNK (9252, CST, 1:1000),P-JNK^Thr183/Tyr185^ (4668, CST, 1:1000),ERK(3372, CST, 1:1000), P-ERK5^Thr218/Tyr220^ (3371, CST, 1:1000), P38MAPK (9212, CST, 1:1000), P-P38MAPK^Thr180/Tyr182^ (4511, CST, 1:1000), β-actin (ab8227; abcam, 1:30000). The membrane was washed three times with TBST for 10 minutes each time. Incubate with HRP-labeled secondary antibody (1:3000) at room temperature for 1 hour, then wash three times with TBST. Finally, develop the protein bands using electrochemiluminescence (ECL) and detect them using enhanced chemiluminescence (GE Healthcare). Analyze the band intensity using ImageJ software.

**Native-PAGE Western blot**

To confirm the integrity of the IL-39(IL23p19/EBI3) heterodimer, non-denaturing polyacrylamide gel electrophoresis (Native-PAGE) followed by Western blot analysis was performed. Unlike SDS-PAGE, which dissociates protein complexes, Native-PAGE preserves the tertiary and quaternary structures of the p19/EBI3 complex, allowing for the detection of the intact heterodimer in its native state. In brief, protein samples were mixed with non-reducing and non-denaturing loading buffer (containing glycerol and bromophenol blue, but lacking SDS). The samples were loaded onto a 4% Tris-Glycine precast gel. Electrophoresis was conducted at 4°C in a running buffer containing 25 mM Tris and 192 mM Glycine (pH 8.3) at a constant voltage of 100 V to minimize heat-induced protein dissociation. Following electrophoresis, proteins were transferred to a polyvinylidene fluoride (PVDF) membrane using a wet transfer system in a buffer devoid of methanol and SDS. The membranes were subsequently blocked with 5% non-fat milk and probed with anti-IL23p19 (MAB1619, R&D) and anti-EBI3 (MAB18341, R&D) antibodies. The observation of immunoblots at the identical molecular weight (approximate 70 kDa) for both antibodies provides compelling evidence for the existence of the IL-39 heterodimer (intact IL23p19/ EBI3 heterodimeric complex).

**Cytokine and hepatic and renal function**

Human plasma aminotransferase (ALT), aspartate aminotransferase (AST), creatinine (Cr), Urea, lactate dehydrogenase (LDH) were assayed according to the protocols of the International Federation of Clinical Chemistry by Hitachi 7600 Fully Automatic Biochemical Analyzer (Hitachi, Tokyo, Japan). The IL-6 (D6050B, R&D), IL-1β (DY201, R&D), TNF-α (DTA00D, R&D; MTA00B, R&D) and IL-10 (D1000B, R&D) levels in human plasma were detected using their respective ELISA kits according to the manufacturer’s instructions. Mouse IL-6 (ab222503, abcam), IL-1β (ab197742, abcam), TNF-α (MTA00B, R&D), IL-10 (ab255729, abcam), ALT (ab282882, abcam), AST (ab263882, abcam) and LDH (AF0216, Beyotime) detected by ELISA kits. Mouse plasma Cr and urea were assayed the protocols of the International Federation of Clinical Chemistry by Hitachi 7600 Fully Automatic Biochemical Analyzer (Hitachi, Tokyo, Japan).

**Isolation and culture of primary murine macrophages**

The methods for isolating and culturing primary mouse macrophages have been described in our previous research[6]. Specifically, mice were euthanized by cervical dislocation and then disinfected by immersion. A scalpel was used to make an incision at the ankle, followed by a longitudinal cut along the leg bone, tearing open the leg skin. The muscles near the groin were removed, and the entire leg was detached along the hip joint. The Achilles tendon was severed, and the major muscles around the tibia were excised. Scissors were used along the femur to remove the major muscles around the femur. The soft tissue of the knee joint was removed to obtain the femur. The fibula and surrounding muscles were severed to expose the complete tibia. The epiphyses at both ends of the femur and tibia were removed, and a 23G needle was used to enter the bone marrow from the distal end. The bone marrow was flushed into cell culture dishes, and approximately 2-3 mL of DMEM was slowly injected into each bone until the bone marrow cavity turned white. DMEM was prevented from flowing back to avoid contamination. The culture medium consisted of DMEM^+^ 10% FBS^+^ 1% penicillin antibiotics + 100 ng/mL M-CSF1. After one day of culture, the cell suspension was transferred to non-treated culture dishes (i.e., smooth culture dishes used for culturing bacteria). On day 4, the culture medium was replaced with fresh medium consisting of DMEM^+^ 10% FBS^+^ 1% antibiotics + 100 ng/mL M-CSF1. On day 7, mature BMDM cells were obtained and >90% of cells were F4/80^+^ macrophages. The cells were scraped off with a scraper for subsequent experiments.

**Preparation of mouse anti-IL39 neutralizing antibody**

Mouse anti-IL39 neutralizing antibody (α-IL-39 nAb) was produced by Sino Biological Inc. (Beijing, China). In short, using rabbit phage display technology to produce murine IL-39-specific monoclonal antibodies. First, heterodimeric IL-39 antigen (composed of IL23p19 and Ebi3 subunits) was expressed in the HEK293 cell system and used in a five-round immunization regimen to induce a strong immune response in rabbits. Immunogenic sequence: N—S4—19-228—GGGSGGGSGGGS —22-196—GGGSGGGSGGGS—His. After reaching optimal serum titers, total RNA was extracted from the spleen and bone marrow of the animals with the strongest immune responses. The antibody variable region gene was amplified by RT-PCR to construct an scFv phage library with a diversity >10⁹ CFU. This library underwent multiple rounds of antigen affinity panning to enrich specific binders. After ELISA screening, at least 10 high-affinity clones were selected for full-length sequence analysis and expression vector construction. Finally, these antibodies were produced using the HEK293 transient transfection system, with each clone yielding 0.1–0.5 mg of purified IgG, demonstrating binding activity against the natural antigen (rm-IL39).

**Immunofluorescent Staining**

Tissues or cells were fixed in 4% paraformaldehyde, permeabilized with 0.2% Triton X-100, and blocked with bovine serum albumin (Servicebio) for 1 h. Primary antibodies against IL23p19 (1:200), EBI3(1:200), GP130 (1:200), F4/80 (1:200), or P38 (1:200) were incubated overnight at 4°C, followed by fluorophore-conjugated secondary antibodies (1:500) and DAPI nuclear counterstaining. Slides were digitized using a Zeiss Axio Scanner Z.1 at 10× magnification. Multiplexed image analysis was conducted via HALO software (Indica Labs). Briefly, regions of interest (ROIs) were manually defined, and tissue segmentation was performed to exclude artifacts and white space.

**Immunoprecipitation**

We screened for IL-39(IL23p19/EBI3)-interacting proteins through immunoprecipitation. The immunoprecipitation (IP) protocol commenced with a necessary pre-clearing phase, where protein lysates were treated with protein A+G agarose beads for 120 minutes at 4°C. This step is critical for reducing background noise in subsequent immunoblotting. After centrifuging the samples at 12,000 rpm to sediment the beads, the resulting supernatant was transferred to fresh tubes and probed with specific primary antibodies under constant end-over-end rotation overnight. To facilitate the solid-phase capture of the targets, the lysates were re-incubated with a secondary volume of protein A+G agarose beads for 2 h. This procedure relies on the high affinity of the beads for the Fc region of the antibodies, effectively concentrating the immunoprecipitated proteins. The beads were then sequestered via brief pulse centrifugation and thoroughly cleansed. This washing process involved five iterations with ice-cold PBS buffer, ensuring that the affinity-purified fraction was free from non-specific cytosolic proteins. The meticulous handling of these steps, particularly the maintenance of cryogenic temperatures, was essential for preserving the native conformation of the captured proteins and their associated binding partners.

**Radioligand binding assay**

GP130-transfected HEK293 cells were cultured for 48 h post-transfection with Lipofectamine 2000. For membrane preparation, cells were homogenized in chilled lysis buffer and centrifuged at 25,000 × g for 45 min at 4°C. The resulting pellet was resuspended in buffer (75 mM Tris, 1 mM EDTA, 12.5 mM MgCl; pH 7.4), with protein concentrations determined via the Bradford method. Binding Studies: In saturation assays, membranes were incubated with UCLA GP130 2(GP130 agonist) at 30°C for 50 min; non-specific binding assay was defined using 1 μM unlabeled UCLA GP130 2. For competition Filtration and Analysis: Reactions were terminated by rapid filtration through GF/C filters using a 24-well FilterMate harvester. Following three chilled washes, dried filters were analyzed via a MicroBeta TriLux scintillation assay. *Kd* values were derived using the Cheng-Prusoff equation. All experiments were performed in triplicate across three independent runs.

**Single-cell RNA sequencing**

According to the method described in our previous study[7]. Specifically, the lung tissues were collected from CLP-induced septic mice at 24 and 48 hours for single-cell sequencing. To remove interference from leukocytes in the blood, 10-20 mL of pre-cooled PBS was slowly perfused through the right ventricle until the lung tissue turned from red to white. The lung lobes were then aseptically removed and placed on ice. The lung tissue was placed in a culture dish and cut into 1 mm³ pieces using surgical scissors. Digestive fluid (usually containing collagenase I/IV and DNase I) was then added. Lung tissue is relatively tough and rich in blood vessels and alveolar cells; therefore, it is recommended to use a constant temperature of 37°C with shaking for 30-45 minutes for digestion. The digested suspension was passed through a 40 μm cell sieve to remove undigested tissue fragments. The solution was then centrifuged at 4°C at low speed and resuspended in PBS containing 0.04% BSA. Cell viability was detected using a fluorescence counte and >85% with no obvious aggregation. Then, the cell concentration was then adjusted to 700-1200 cells/ml for test. The single-cell suspension was partitioned into GEMs using the Chromium Controller, where mRNA was captured and reverse-transcribed into cDNA. Following PCR amplification and purification, the products were into single-stranded circular DNA and replicated via rolling circle amplification to produce DNA nanoballs (DNBs). These high-quality DNBs were then loaded onto nanoarrays for sequencing using combinatorial probe anchoring synthesis (cPAS) on the DNBSEQ platform (OE Biotechnology Co., Ltd., Shanghai, China).

**scRNA-seq data processing and analysis**

Single-cell RNA-seq Data Processing: Initial processing of raw sequencing data was performed using Cell Ranger (v6.0.0). Downstream analysis was conducted using the Seurat R package and Python-based tools. During quality control (QC), cells were excluded if they expressed fewer than 200 genes or if their gene count exceeded 90% of the maximum observed distribution to mitigate potential artifacts. DoubletFinder (or similar algorithms) was employed to identify and remove potential doublets. Normalization and Clustering: The gene expression matrix was normalized, followed by the identification of the top 2,000 highly variable genes (HVGs) for Principal Component Analysis (PCA). Unsupervised clustering was visualized in two dimensions using t-distributed Stochastic Neighbor Embedding (t-SNE). Cell Type Annotation and Differential Expression: For each cluster, cluster-specific marker genes were identified using the FindAllMarkers function (logFC > 0.25, min.pct > 0.1, and Go enrichment was calculated using the phyper function in R, and GO terms with a False Discovery Rate (FDR) ≤0.05 were considered significantly enriched.

**Proteomics and analyses**

Mouse primary macrophages were stimulated with LPS and treated with rm-IL-39 or a solvent control. Cells were collected and lysed at 4°C with 8 M urea lysis buffer. During protein extraction, disulfide bonds were reduced with dithiothreitol (DTT) and alkylated with iodoacetamide (IAA). Serial digestion was performed, first with Lys-C for 2 hours, followed by trypsin digestion overnight. TMT labeling and LC-MS/MS: For relative quantification, 200 μg of protein in each sample was labeled with a TMT 10-fold isotope tag. Labeled peptides were analyzed by high-resolution LC-MS/MS using a data-dependent acquisition (DDA) strategy. Raw data were processed using the MaxQuant environment, and peptide and protein identification was automated using the Andromeda search engine. To ensure high-confidence quantification, a localization probability threshold of ≥0.75 was applied. Bioinformatics and Functional Annotation: We identified differentially expressed proteins (DEPs) based on normalized fold changes and p-value criteria. Functional characterization included gene ontology (GO), orthologous gene clusters (COG), and KEGG pathway annotation. To elucidate the systemic effects of IL-39 on the macrophage proteome, we performed advanced analyses including protein-protein interaction (PPI) networks (via the STRING database).

**Identification of IL-39 interacting proteins by LC-MS/MS**

To identify the interacting proteins with IL-39(IL23p19/EBI3), RAW264.7 cells were transiently transfected with either FLAG-tagged IL-39(IL23p19/EBI3) expression plasmids or a FLAG-empty vector control for 24 hours using Lipofectamine 3000. Following incubation, cells were harvested and lysed in a chilled NP-40 lysis buffer supplemented with a comprehensive protease and phosphatase inhibitor cocktail to preserve protein-protein interactions. Co-Immunoprecipitation (Co-IP): The clarified whole-cell lysates were subjected to affinity purification by incubation with anti-FLAG M2 magnetic beads (or agarose) overnight at 4°C with gentle rotation. After several stringent washes with lysis buffer to eliminate non-specific binding, the immuno-complexes were eluted using competitive 3×FLAG peptide or acidic elution buffer. Mass Spectrometry and Data Analysis: The eluted proteins were processed via in-solution trypsin digestion and subsequently analyzed by liquid chromatography-tandem mass spectrometry (LC-MS/MS) performed by Genechem (Shanghai, China). Raw MS data were searched against the Mus musculus database. To filter for high-confidence candidates, proteins identified in the FLAG-IL-39(IL23p19/EBI3) group were compared against the vector control. Candidates were selected based on a fold-change enrichment > 2.0 and a unique peptide count >2. This integrated approach allowed for the identification of potential intracellular binding proteins involved in IL-39-mediated signaling.

**Surface plasmon resonance**

Serial concentrations of recombinant IL-39 (0.001 μM, 0.01 μM, 0.1 μM, 1 μM, 10 μM and 100 μM) were flowed over GP130-coupled sensor chips. The association phase was recorded from 0 s to 50 s, followed by dissociation monitoring for an additional 100 s with running buffer injection. Sensorgrams exhibited obvious concentration-dependent binding responses during association and gradual dissociation after buffer washing. Global fitting based on the 1:1 Langmuir binding model yielded an equilibrium dissociation constant (KD).

**Bacterial burdens**

Peritoneal lavage fluid (PLF) and blood samples were harvested from CLP mice to assess bacterial burden. After performing serial dilutions, the samples were cultured on Luria-Bertani (LB) agar and blood agar (5% sheep blood, Thermo Fisher Scientific). Cultures were maintained at 37°C for 24 hours, followed by colony counting to determine the degree of systemic and local infection.

**References**

[1]	a) S. Mervyn, D. Clifford S, S. Christopher Warren, S.-H. Manu, A. Djillali, B. Michael, B. Rinaldo, B. Gordon R, C. Jean-Daniel, C. Craig M, H. Richard S, L. Mitchell M, M. John C, M. Greg S, O. Steven M, R. Gordon D, v. d. P. Tom, V. Jean-Louis, A. Derek C, *JAMA* **2016**, *315* (8), https://doi.org/10.1001/jama.2016.0287; b) C. Huan, Z. Huali, W. Kangkai, Y. Yongming, X. Xianzhong, *Zhonghua Wei Zhong Bing Ji Jiu Yi Xue* **2020**, *31* (11), https://doi.org/10.3760/cma.j.issn.2095-4352.2019.11.001.

[2]	Z. Wen-Wu, W. Shun-Shun, D. Yang-Dong, W. Xin-Yi, C. Ting, G. Ye, J. Sheng-Wei, Z. Pu-Hong, *Free Radic. Biol. Med.* **2024**, *215* (0), https://doi.org/10.1016/j.freeradbiomed.2024.02.026.

[3]	B. Haobo, L. Qian, W. Chunxiang, X. Fang, L. Jiayu, W. Ke, D. Hao, Y. Yibing, L. Yi, L. Xiaofei, C. Ju, *Sci. Transl. Med.* **2024**, *16* (732), https://doi.org/10.1126/scitranslmed.adi3275.

[4]	a) M. Deng, Y. Tang, W. Li, X. Wang, R. Zhang, X. Zhang, X. Zhao, J. Liu, C. Tang, Z. Liu, Y. Huang, H. Peng, L. Xiao, D. Tang, M. Scott, Q. Wang, J. Liu, X. Xiao, S. Watkins, J. Li, H. Yang, H. Wang, F. Chen, K. Tracey, T. Billiar, B. Lu, *Immunity* **2018**, *49* (4), 740, https://doi.org/10.1016/j.immuni.2018.08.016; b) J. Gu, L. Luo, Q. Wang, S. Yan, J. Lin, D. Li, B. Cao, H. Mei, B. Ying, L. Bin, F. G. Smith, S. W. Jin, *Laboratory investigation; a journal of technical methods and pathology* **2018**, *98* (6), 715, https://doi.org/10.1038/s41374-018-0031-x.

[5]	a) A. Parisa, J. Atousa, H. Maryam, T. Soroush, D. David, M. Negin, *Cytokine* **2025**, *192* (0), https://doi.org/10.1016/j.cyto.2025.156969; b) M. Izuru, O. Mio, H. Hideaki, C. Yukino, O. Naoko, I. Shinya, K. Chiaki, X. Mingli, S. Katsuko, F. Koji, K. Masahiko, H. Shin-Ichi, M. Kouji, Y. Takayuki, *J. Clin. Invest.* **2020**, *130* (11), https://doi.org/10.1172/jci122732.

[6]	Z. Sisi, M. Minqi, L. Zhongwang, H. Yu, L. Hui, F. Panhan, J. Shengwei, *FASEB J.* **2020**, *34* (9), https://doi.org/10.1096/fj.202000665RR.

[7]	W. Hui, H. Panwei, W. Zhenxia, T. Chao, L. Chuanlong, L. Xiangyu, Y. Tao, Q. Yang, L. Sunwang, L. Hanzhi, W. Gan, L. Yan, W. Jianguang, J. Shengwei, *Cell Rep* **2025**, *44* (4), https://doi.org/10.1016/j.celrep.2025.115469.

**2. Supplementary figures**

**Figure S1. Single-cell transcriptional profiling of lung tissue cells from sepsis mice**

(A) The lung tissue obtained from septic mice at 24 or 48 hours after CLP was analyzed by scRNA-seq. t-distributed stochastic neighbor embedding (t-SNE) plot showing the unsupervised clustering (sham: n = 3, CLP: n = 3). (B) t-Distributed stochastic neighbor embedding plot displaying the major cell types by molecular markers. (C) Density maps show the difference in cell numbers between the sham and sepsis groups. (D) Bubble plot showing expression of the top 16 differentially signature genes (DEGs; color scaled) across each clusters. (E) Immunofluorescence staining of leukocytes (CD45, red) and IL-39 (EBI3 subunit, green) in tissues from CLP mice. DAPI (blue) was used to stain nuclei.

**Figure S2. IL-39 treatment deteriorates sepsis-induced severity and mortality in mice.**

(A) Various doses of rmIL-39 (0.125, 0.25, and 0.5 μg/g) were injected intravenously (*i.v.*) into male mice (n = 20 per group) immediately after CLP, and survival was monitored for 72 hours. (B) Male mice (n = 20 per group) were injected intravenously (*i.v.*) with rmIL-39 (0.5 μg/g) at 0, 6, and 12 hours after CLP, and survival was monitored for 72 hours. (C) Both male and female mice (n = 20 per group) were injected intravenously (*i.v.*) with rmIL-39 (0.5 μg/g) immediately after CLP, and survival was monitored for 72 hours. (D) Male mice were injected intravenously (*i.v.*) with rmIL-39 (0.5μg/g) immediately after CLP in addition to antibiotic (imipenem-cilastatin, 25 mg/kg) administration at 8, 16, and 24 hours after CLP, and survival was monitored for 72 hours (CLP+PBS, n=20; CLP+ rmIL-39, n=22). (E) Serological markers of organ injury, including AST, ALT, LDH, creatinine (CR), and Urea in septic mice (n = 8 per group) injected with or without rmIL-39 (0.5 μg/g) at 24 hours after CLP. (F) Representative examples of hematoxylin and eosin-stained heart, liver, lung, and kidney tissues from mice (n = 8 per group). Scale bars, 100 μm. Histological scores were determined by two independent investigators. (G) Neutrophil (Ly6G+) accumulation in tissues were observed by Immunohistochemistry. (H) Serological markers of pro-inflammatory factors (IL-6, IL-1β, TNF-α) and anti-inflammatory factor (IL-10) were detected by ELISA. Data are represented as mean ± SD and determined by Kruskal-Wallis test followed by the two-tailed unpaired Student’s t test in (E, G, H). Data are represented as Median (Quartile) [M (P25, P75)] (F) and analyzed by using the Kruskal-Wallis test. Kaplan- Meier analysis followed by log-rank test in (A to D).

**Figure S3. Macrophage-specific silencing of IL-39 alleviates sepsis-induced multiple organ injury and mortality in mice.**

(A) Schematic diagram of AAV-mediated macrophage-specific silencing of IL-39(IL23p19/EBI3) experiment in mice. Four-week-old male WT mice were treated with AAV-Lyz2-sh IL-39(IL23p19/EBI3) or AAV-GFP-shRNA by tail vein injection. After 4 weeks of AAV injection, all mice underwent either CLP or sham. (B) Macrophage IL-39 protein levels in mice treated with or without AAV-Lyz2-shIL-39, detected by Native-PAGE Western blot (n=3).

(C) The survival was monitored for 72 hours after CLP (CLP+AAV-GFP-shRNA, n=19; CLP+AAV-Lyz2-sh IL-39, n=21). (D) Serological markers of organ injury, including AST, ALT, LDH, creatinine (Cr), urea from CLP mice (n = 8 per group). (E) Representative examples of hematoxylin and eosin-stained heart, liver, lung, and kidney tissues. Scale bars, 100 μm. Histological scores. (F) Neutrophil (Ly6G^+^) accumulation in tissues. (G) Serological markers of pro-inflammatory factors (IL-6, IL-1β, TNF-α) and anti-inflammatory factor (IL-10). Data are represented as mean ± SD and determined by Kruskal-Wallis test followed by the two-tailed unpaired Student’s t test in (D, F, G). Data are represented as Median (Quartile) [M (P25, P75)] (E) and analyzed by using the Kruskal-Wallis test. Kaplan- Meier analysis followed by log-rank test in (C).

 **Figure S4. Generation and physiological characterization of macrophage-specific IL-39 (Ebi3 subunit) deficient mice**

(A) The construction strategy of Macrophage-specific IL-39 (Ebi3 subunit) knockout in mice. (B) Representative polymerase chain reaction (PCR) genotyping of Ebi3^flox/flox^ and Lyz2-Cre mice. Genotyping primers for Ebi3^flox/flox^ mice were described as follows: F1 (5’- CACCCTTGAACTTAGACATTTGTGG) and R1 (AAGAACTCCCTAGACTACCCACACC). The length of the PCR product of WT primers was 320bp and Ebi3 ^flox/flox^ primers was 425 bp. Genotyping primers for Lyz2-Cre mice were described as follows: F1 (5’- GCACACAGCTCAAATGTAGGAAACC-3’) and R1 (5’- ACACAGCATTGGAGTCAGAAGGG-3’). The length of PCR product of Lyz2-Cre primers was 482 bp. (C) Protein expression level of Ebi3 and IL-39 in macrophage, detected by Native-PAGE Western blot. (D) Serological markers of organ function, including AST, ALT, LDH, creatinine (Cr) and urea from control mice (n = 8 per group). (E) Representative examples of hematoxylin and eosin-stained heart, liver, lung, and kidney tissues. Scale bars, 100 μm. Histological scores.

**Figure S5. Macrophage-specific IL-39 (Ebi3 subunit) deficiency alleviates sepsis-induced multiple organ injury and mortality in mice.**

(A) Schematic diagram of macrophage-specific IL-39 (Ebi3 subunit) deficiency experiment. (B) The survival was monitored for 72 hours after CLP (Ebi3^WT^, n=20; Ebi3^MKO^, n=21). (C) Serological markers of organ injury, including AST, ALT, LDH, creatinine (Cr), urea from CLP mice (n = 8 per group). (D) Representative examples of hematoxylin and eosin-stained heart, liver, lung, and kidney tissues. Scale bars, 100 μm. Histological scores. (E) Neutrophil (Ly6G^+^) accumulation in tissues. (F) Serological markers of pro-inflammatory factors (IL-6, IL-1β, TNF-α) and anti-inflammatory factor (IL-10).

**Figure S6. Deficiency of macrophage GP130 eliminated the protection of IL-39 deficiency against sepsis**

(A) Schematic diagram of AAV-mediated macrophage-specific silencing of GP130 in macrophage-specific Ebi3 (a subunit of IL-39) deficiency mice (Ebi3^MKO^). Four-week-old male Ebi3^MKO^/Ebi3^WT^ mice were treated with AAV-Lyz2-sh GP130 by tail vein injection. After 4 weeks of AAV injection, all mice underwent CLP. (B) The protein expression of Ebi3, IL-39, GP130 levels in macrophage are detected by Native-PAGE Western blot. (C) The survival was monitored for 72 hours after CLP (n = 20 per group). (D) Serological markers of organ injury, including AST, ALT, LDH, creatinine (Cr), urea from CLP mice (n = 8 per group). (E) Representative examples of hematoxylin and eosin-stained heart, liver, lung, and kidney tissues and histological scores. Scale bars, 100 μm. (F) Neutrophil (Ly6G^+^) accumulation in tissues. (G) Serological markers of pro-inflammatory factors (IL-6, IL-1β, TNF-α) and anti-inflammatory factor (IL-10). Data are represented as mean ± SD and determined by Kruskal-Wallis test followed by the two-tailed unpaired Student’s t test in (D, G, F). Data are represented as Median (Quartile) [M (P25, P75)] (E) and analyzed by using the Kruskal-Wallis test. Kaplan- Meier analysis followed by log-rank test in (C).

**Figure S7. Validation of macrophage-specific silencing/overexpression of IL-39**

(A) Flow cytometry gating scheme for IL-39⁺macrophages. Single live macrophages (CD45⁺F4/80⁺) were sequentially gated, followed by identification of IL-39^+^macrophages via detection of constituent subunits EBI3 and IL-23p19. (B) Four-week-old male WT mice were treated with AAV-Lyz2-shIL-39(IL23p19/EBI3) or AAV-GFP-shRNA by tail vein injection. After 4 weeks of AAV injection, IL-39^+^macrophages counts were determined by flow cytometry (n=3). (C) Four-week-old male WT mice were treated with AAV-Lyz2-IL-39(IL23p19/EBI3) or AAV-GFP-RNA by tail vein injection. After 4 weeks of AAV injection, IL-39^+^macrophages counts were determined by flow cytometry. (D,E) IL-39(IL23p19/EBI3) expression in BMDM was determined by immunofluorescence. (F) Flow sorting of B cells and T cells. Sequential gating was performed to isolate intact single live CD45⁺ leukocytes for subsequent separation and collection of T and B cell subsets. (G,H) IL-39 expression in B cells and T cells were detected by Native-PAGE Western blot (n=3).

**Figure S8. IL-39-mediated inflammation rely on p38 activation, related to Figure 7**

Primary macrophages were treated with rmIL-39/PBS and SB203580(p38-specific inhibitor SB203580)/DMSO, stimulated with 100 ng/mL LPS for 60 min, then total cellular protein was harvested to quantify IL-6, IL-1β and TNF-α expression via ELISA.

**Figure S9. anti-IL-39 neutralizing antibody(α-IL-39 nAb) inhibit the activation of MAPK and the subsequent production of inflammatory factors, related to Figure 7**

(A) Primary macrophages were treated with α-IL-39 nAb/PBS, stimulated with 100 ng/mL LPS for 0,10,30,60 min, then total cellular proteins were extracted for detecting the activation of MAPK (including phospho-c-Jun, phospho-JNK, phospho-Erk, phospho-p-38) by Western blot analysis (n=3). (B) Pro-inflammatory factors (IL-6, IL-1β, TNF-α) in macrophages lysates were detected by ELISA (n=3).

**Figure S10. IL-39 inhibits the bacterial phagocytic function of macrophages during sepsis**

(A) Serial dilutions were prepared from peritoneal lavage fluid (PLF) and blood samples collected from rmIL-39-treated and anti-IL-39 neutralizing antibody (α-IL-39 nAb)-treated mice 24 hours after CLP. Representative culture plates for PLF and blood from 3 independent experiments. (B) Phagocytosis of *E.coli* by bone marrow–derived macrophages (BMDM) isolated from rmIL-39-treated and anti-IL-39 neutralizing antibody (α-IL-39 nAb)-treated mice 24 hours after CLP. Images display representative Luria-Bertani (LB) culture plates.

**Figure S11. The rescue of IL-39 abolishes the protective effects of IL-39 deficiency for murine sepsis.**

(A) Schematic diagram of macrophage-specific IL-39 (Ebi3 subunit) deficiency were treated with AAV-Lyz2-IL-39(IL23p19/EBI3) or AAV-GFP-RNA by tail vein injection. After 4 weeks of AAV injection, all mice underwent CLP. (B) Macrophage IL-39 protein levels in Ebi3^MKO^ mice treated with or without AAV-Lyz2-IL-39(IL23p19/EBI3), detected by Native-PAGE Western blot (n=3). (C) The survival was monitored for 72 hours after CLP (n = 10 per group). (D) Serological markers of organ injury, including AST, ALT, LDH, creatinine (Cr), urea from CLP mice (n = 5 per group). (E) Representative examples of hematoxylin and eosin-stained heart, liver, lung, and kidney tissues. Scale bars, 100 μm. Histological scores. (F) Neutrophil (Ly6G^+^) accumulation in tissues detected by immumohistochemical staining. (G) Serological markers of pro-inflammatory factors (IL-6, IL-1β, TNF-α) and anti-inflammatory factor (IL-10). Data are represented as mean ± SD (D,F,G) and determined by the two-tailed unpaired Student’s t test. Data are represented as Median (Quartile) [M (P25, P75)] (E) and analyzed by using the Kruskal-Wallis test. Kaplan- Meier analysis followed by log-rank test in (C).

**3. Supplementary tables**

| **Table S1. Clinical characteristics of sepsis patients in the discovery cohort (Anhui)** | | | | |
| --- | --- | --- | --- | --- |
| **Characteristics** | **Healthy control**  (n=66) | **Patient control** (n=65) | **Sepsis patients** | |
|  |  |  | **no-shock** (n=47) | **shock** (n=43) |
| Age, y | 63(52-75) | 67(26-84) | 67(22-82) | 69(19-85) |
| Male | 43(65.15%) | 41(63.07%) | 29(61.70%) | 28(65.12%) |
| Smoking history | 13(19.70%) | 11(16.92%) | 9(19.15%) | 10(23.26%) |
| Drinking history | 7(10.61%) | 5(7.69%) | 4(8.51%) | 3(6.98%) |
| Complication（ARDS） | N/A | 1(1.54%) | 7(14.89%) | 10(23.26%) |
| Comorbidities Hypertension | N/A | 18(27.69%) | 14(29.79%) | 17(39.53%) |
| Diabetes mellitus | N/A | 21(32.31%) | 12(25.53%) | 13(30.23%) |
| Chronic cardiovascular disease | N/A | 8(12.31%) | 9(19.15%) | 12(27.91%) |
| Cerebrovascular disease | N/A | 9(12.85%) | 7(14.89%) | 6(13.95%) |
| Peripheral vascular disease | N/A | 5(7.69%) | 8(17.02%) | 5(11.63%) |
| Chronic pulmonary disease | N/A | 15(23.08%) | 10(21.28%) | 10(23.26%) |
| Chronic liver disease | N/A | 19(29.23%) | 12(25.53%) | 14(32.56%) |
| Chronic kidney disease | N/A | 18(27.69%) | 9(19.15%) | 12(27.91%) |
| **Type of pneumonia** |  |  |  |  |
| CAP | N/A | N/A | 34(72.34%) | 28(65.12%) |
| HAP | N/A | N/A | 3(6.38%) | 6(13.95%) |
| Non | N/A | N/A | 10(21.28%) | 9(20.93%) |
| **Isolates, no. of patients** |  |  |  |  |
| Gram-positive | N/A | N/A | 10(21.28%) | 8(18.60%) |
| Gram-negative | N/A | N/A | 24(51.06%) | 21(48.84%) |
| Fungus | N/A | N/A | 8(17.02%) | 5(11.63%) |
| Miscellaneous | N/A | N/A | 5(10.64%) | 9(20.93%) |
| Ulinastatin | N/A | N/A | 5(10.64%) | 7(16.28%) |
| Heparin Sodium | N/A | N/A | 39(82.98%) | 41(95.35%) |
| Low Molecular Weight Heparin | N/A | N/A | 26(55.32%) | 24(55.81%) |
| CRRT | N/A | N/A | 13(27.66%) | 19(44.18%) |
| Vasopressors | N/A | N/A | 27(57.45%) | 37(86.04%) |
| Blood Transfusion | N/A | N/A | 24(51.06%) | 29(67.44%) |
| ECMO | N/A | N/A | 4(8.51%) | 9(20.93%) |
| **Laboratory indicators** |  |  |  |  |
| WBC(10*9/L) | 5.5(3.26-10.73) | 12.4(1.97-24.59) | 14.76(2.7-35.2) | 15.3(3.8-39.6) |
| NEU(10*9/L) | 4.21(1.4-8.5) | 9.93(1.1-24.5) | 11.32(2.5-26) | 12.5(1.5-47.7) |
| IL-6(pg/ml) | N/A | 52.33(22.63-156.27) | 54.29(21.43-152.4) | 223.86(42.5-573.32) |
| CRP(mg/L) | 3.87(0.17-5.22) | 49.42(1.88-138.58) | 85.3(1.23-268.01) | 164.53(15.92-351.85) |
| PCT(ng/ml) | 0.27(0.03-1.16) | 0.35(0.04-0.659) | 17.42(0.034-25.22) | 38.72(1.75-75.0) |
| INR | N/A | 0.97(0.81-1.12) | 1.09(0.84-1.42) | 1.32(1.18-1.56) |
| Lac(mmol/L) | N/A | 1.17(0.76-1.51) | 1.3(0.58-2.33) | 2.41(1.22-6.2) |
| IL-39(pg/ml) | 23.93(6.12-41.02) | 24.76(15.20-36.55) | 29.23(11.65-52.9) | 44.07(22.3-65.05) |
| **Severe disease score** |  |  |  |  |
| GCS | N/A | 15(13-15) | 14(10-15) | 11(4-15) |
| SOFA | N/A | 2(0-4) | 6(1-12) | 13(5-18) |
| APACHE Ⅱ | N/A | 8(4-12) | 16(11-24) | 24(15-29) |

| **Table S2. Clinical characteristics of sepsis patients in the validation cohort (Zhejiang)** | | | | |
| --- | --- | --- | --- | --- |
| **Characteristics** | **Healthy control**  (n=104) | **Patient control** (n=99) | **Sepsis patients** | |
|  |  |  | **no-shock** (n=68) | **shock** (n=54) |
| Age, y | 61(32-77) | 65(21-91) | 63(16-88) | 64(27-89) |
| Male | 69(66.35%) | 60(60.61%) | 42(61.76%) | 34(62.96%） |
| Smoking history | 16(15.38%) | 11(11.11%) | 12(17.65%) | 7(12.96%) |
| Drinking history | 5(4.80%) | 2(2.02%) | 3(4.41%) | 2(3.70%) |
| Complication(ARDS) | N/A | 2(2.02%) | 9(13.86%) | 15(27.78%) |
| Comorbidities Hypertension | N/A | 36(36.36%) | 26(38.6%) | 27(50.00%) |
| Diabetes mellitus | N/A | 18(18.18%) | 8(11.88%) | 13(24.07%) |
| Chronic cardiovascular disease | N/A | 15(15.15%) | 13(18.81%) | 13(24.07%) |
| Cerebrovascular disease | N/A | 10(10.10%) | 12(17.65%) | 9(16.67%) |
| Peripheral vascular disease | N/A | 6(6.06%) | 1(1.47%) | 2(3.70%) |
| Chronic pulmonary disease | N/A | 7(7.07%) | 4(5.88%) | 4(7.40%) |
| Chronic liver disease | N/A | 10(10.10%) | 5(7.35%) | 6(11.11%) |
| Chronic kidney disease | N/A | 19(19.19%) | 13(19.12%) | 14(25.93%) |
| **Type of pneumonia** |  |  |  |  |
| CAP | N/A | N/A | 28(41.58%) | 31(57.41%) |
| HAP | N/A | N/A | 4(5.94%) | 4(7.41%) |
| Non | N/A | N/A | 36(52.48%) | 19(35.19%) |
| **Isolates, no. of patients** |  |  |  |  |
| Gram-positive | N/A | N/A | 15(22.06%) | 7(12.96%) |
| Gram-negative | N/A | N/A | 33(48.53%) | 35(64.81%) |
| Fungus | N/A | N/A | 4(5.88%) | 3(5.56%) |
| Miscellaneous | N/A | N/A | 16(23.53%) | 9(16.67%) |
| Ulinastatin | N/A | N/A | 5(6.93%) | 9(16.67%) |
| Heparin Sodium | N/A | N/A | 51(75.24%) | 50(92.59%) |
| Low Molecular Weight Heparin | N/A | N/A | 42(62.38) | 38(70.37%) |
| CRRT | N/A | N/A | 29(42.57%) | 31(57.41%) |
| Vasopressors | N/A | N/A | 48(70.30%) | 44(81.48%) |
| Blood Transfusion | N/A | N/A | 31(45.54%) | 31(57.41%) |
| ECMO | N/A | N/A | 8(11.88%) | 4(7.41%) |
| **Laboratory indicators** |  |  |  |  |
| WBC(10*9/L) | 6.68(2.01-14.53) | 10.34(2.07-22.5) | 12.2(1.2-35.4) | 14.3(2.5-42.7) |
| NEU(10*9/L) | 3.88(1.37-9.57) | 9.36(1.22-29.3) | 9.8(1.7-22.2) | 10.33(1.43-47.8) |
| IL-6(pg/ml) | N/A | 49.57(22.53-197.43) | 62.88(23.14-159.55) | 247.43(48.53-526.3) |
| CRP(mg/L) | 2.98(0.11-4.86) | 58.26(0.5-138.58) | 93.55(1.02-268.98) | 102.42(12.42-363.34) |
| PCT(ng/ml) | 0.31(0.07-0.96) | 1.34(0.31-3.62) | 2.67(0.035-8.3) | 7.23(1.39-72.34) |
| INR | N/A | 1.01(1.05-1.20) | 1.22(0.98-1.52) | 1.34(1.13-1.67) |
| Lac(mmol/L) | N/A | 0.85(0.89-1.56） | 1.56(0.26-2.04） | 2.04(1.15-2.91） |
| IL-39(pg/ml) | 25.37(15.1-49.3) | 28.31(21.8-37.25) | 29.07(7.76-78.16) | 43.38(23.28-78.16) |
| **Severe disease score** |  |  |  |  |
| GCS | N/A | 15(14-15) | 14(9-15) | 11(6-15) |
| SOFA | N/A | 1(0-3) | 5(2-11) | 12(4-21) |
| APACHE Ⅱ | N/A | 5(2-11) | 14(13-24) | 26(16-35) |
